# The therapeutic efficacy of different configuration nano-polydopamine drug carrier systems with photothermal synergy against head and neck squamous cell carcinoma

**DOI:** 10.1093/rb/rbae073

**Published:** 2024-06-20

**Authors:** Yuhao Guo, Bo Li, Huixu Xie, Chenzhou Wu, Guixue Wang, Kexin Yao, Longjiang Li

**Affiliations:** State Key Laboratory of Oral Diseases, National Center for Stomatology, National Clinical Research Center for Oral Diseases, West China Hospital of Stomatology, Sichuan University, Chengdu 610041, China; Department of Stomatology, Xinqiao Hospital of Army Medical University, Chongqing 400037, China; State Key Laboratory of Oral Diseases, National Center for Stomatology, National Clinical Research Center for Oral Diseases, West China Hospital of Stomatology, Sichuan University, Chengdu 610041, China; Department of Orthodontics, West China Hospital of Stomatology, Sichuan University, Chengdu 610041, China; State Key Laboratory of Oral Diseases, National Center for Stomatology, National Clinical Research Center for Oral Diseases, West China Hospital of Stomatology, Sichuan University, Chengdu 610041, China; Department of Head and Neck Oncology, West China Hospital of Stomatology, Sichuan University, Chengdu 610041, China; State Key Laboratory of Oral Diseases, National Center for Stomatology, National Clinical Research Center for Oral Diseases, West China Hospital of Stomatology, Sichuan University, Chengdu 610041, China; Department of Head and Neck Oncology, West China Hospital of Stomatology, Sichuan University, Chengdu 610041, China; State and Local Joint Engineering Laboratory, Bioengineering College of Chongqing University, Chongqing 400044, China; Multi-Scale Porous Materials Center, Institute of Advanced Interdisciplinary Studies, School of Chemistry and Chemical Engineering, Chongqing University, Chongqing 400044, China; State Key Laboratory of Oral Diseases, National Center for Stomatology, National Clinical Research Center for Oral Diseases, West China Hospital of Stomatology, Sichuan University, Chengdu 610041, China; Department of Head and Neck Oncology, West China Hospital of Stomatology, Sichuan University, Chengdu 610041, China

**Keywords:** nano-polydopamine (nPDA), photothermal effect, thermo-chemotherapy, head and neck squamous cell carcinoma (HNSCC), drug carrier system

## Abstract

Head and neck squamous cell carcinoma (HNSCC) is the sixth most common malignant tumor worldwide. Considering its special anatomical site and the progressive resistance to chemotherapy drugs, the development of more effective, minimally invasive and precise treatment methods is urgently needed. Nanomaterials, given their special properties, can be used as drug carrier systems to improve the therapeutic effect and reduce the adverse effects. The drug carrier systems with photothermal effect can promote the killing of cancer cells and help overcome drug resistance through heat stress. We selected dopamine, a simple raw material, and designed and synthesized three different configurations of nano-polydopamine (nPDA) nanomaterials, including nPDA balls, nPDA plates and porous nPDA balls. In addition to the self-polymerization and self-assembly, nPDA has high photothermal conversion efficiency and can be easily modified. Moreover, we loaded cisplatin into three different configurations of nPDA, creating nPDA-cis (the nano-drug carrier system with cisplatin), and comparatively studied the properties and antitumor effects of all the nPDA and nPDA-cis materials *in vitro* and nPDA-cis *in vivo*. We found that the photothermal effect of the nPDA-cis balls drug carrier system had synergistic effect with cisplatin, resulting in excellent antitumor effect and good clinical application prospects. The comparison of the three different configurations of drug carrier systems suggested the importance of optimizing the spatial configuration design and examining the physical and chemical properties in the future development of nano-drug carrier systems. In this study, we also noted the duality and complexity of the influences of heat stress on tumors *in vitro* and *in vivo*. The specific mechanisms and the synergy with chemotherapy and immunotherapy will be an important research direction in the future.

## Graphical abstract

**Figure:**
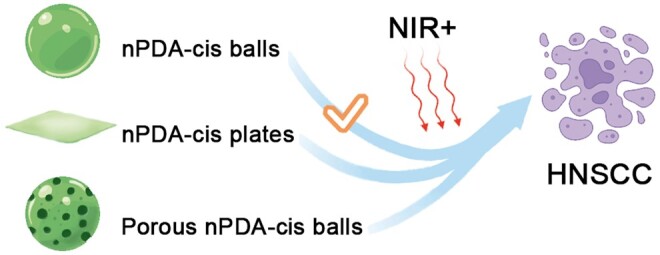

## Introduction

Head and neck squamous cell carcinoma (HNSCC) is the sixth most prevalent cancer in the world. China has the third-highest incidence of oral and lip cancer and second highest number of deaths in the world [1]. At present, surgery, radiotherapy and chemotherapy are the main treatment options in clinical practice. However, considering the specific anatomical site of HNSCC, surgical treatment can have an impact on the patient’s voice, eating, appearance and psychology. In the meantime, resistance to chemotherapy treatment drugs is becoming an increasing problem. Therefore, more precise, minimally invasive and effective treatments are urgently needed, and the combination of new treatments (e.g. heat therapy and immunotherapy) with standard treatments (radiotherapy, chemotherapy and surgical treatment) is beginning to receive attention [2–4].

Heat therapy is a non-invasive treatment that can target cancer cells. In general, cancer cells exhibit different susceptibility to cell death induced by heat compared with normal cells. Exposure to the same thermal dose (defined by exposure time and given temperature) is more likely to induce a cell death outcome in cancer cells. Therefore, appropriate heat stress can promote the killing of tumor cells without causing damage to normal cells [5]. It has also been suggested that heat therapy enhances the efficacy of radiotherapy or chemotherapy mainly by activating the immune system [6]. Therefore, utilizing the synergistic effect of chemotherapy and hyperthermia has become an important direction for the development of new drugs [7, 8]. Besides, in oral cancer, local heat application can reduce the trauma produced by the treatment [9, 10].

Cisplatin is a classical chemotherapeutic agent that is commonly used in clinical practice, but defects in apoptosis-related pathways and some other mechanisms may lead to cisplatin resistance. Therefore, addressing drug resistance has become an important research topic to enhance the efficacy of cisplatin therapy in clinical practice [11]. Previous studies have shown that thermotherapy can synergize with cisplatin to reduce drug resistance, decrease cancer cell survival and enhance therapeutic efficacy [12]. In addition, thermotherapy can reverse the drug resistance in tumors that have already developed cisplatin resistance [13]. Thus, thermotherapy has a positive effect on cancer treatment as an adjunct to radiotherapy and chemotherapy. The key to applying thermotherapy or thermochemotherapy in oncology is to ensure safe, effective and stable local heat application on the tumor.

Nanomaterials are considered drug carrier systems with great potential due to their abilities of increasing the water solubility of hydrophobic drugs, protecting the active sites of antitumor drugs and changing the distribution and metabolic pattern of drugs in the human body, thereby improving the therapeutic index and reducing adverse effects [14]. With the advancement of nanotechnology, a variety of nanomaterials with photothermal effects have been developed to provide a useful tool for the synergy of thermotherapy and chemotherapy in the treatment of tumors [15]. Meanwhile, the encapsulation, modification and transport effects of nanomaterials for drugs can well reduce the off-target toxicity of chemotherapeutic drugs and increase the tissue accumulation and retention of cisplatin [16].

Polydopamine (PDA) is a novel polymeric material formed by oxidative polymerization and self-assembly of dopamine, originally used to enhance cell adhesion [17]. It has recently been applied in the biomedical field because of the simple ingredients and easy synthesis process [18]. It also shows good biocompatibility, easy functional modification characteristics and excellent photothermal conversion efficiency. The possibility of using PDA in photothermal therapy in the biomedical field has received attention in recent years [19]. Many studies have shown that structure has an important influence on function in living organisms. For example, in pharmaceutical research, it has been found that drugs with different chirality have different biological activities and toxic effects [20]; at the molecular level, the functions exercised by biomolecules are often related to their configurations [21]. The same phenomenon exists in nanomaterials, where nanomaterials synthesized from the same monomer can form different microconfigurations with different properties, and thus, be used in different fields [22–24]. There have been some studies on the differentiation or proliferation of cells on scaffolds with different morphological features [25]. When it comes to drug carrier systems, however, comparative studies on the physicochemical properties of different configurations of nanomaterials synthesized from the same monomer, and the biological effects and other properties under the same experimental conditions are still relatively rare [26].

In this study, we designed and successfully synthesized three different configurations of nano-PDA (nPDA), namely, nPDA balls, nPDA plates and porous nPDA balls. These were then loaded with cisplatin, thereby forming three nano-PDA-cisplatin drug carrier systems (nPDA-cis). A comparative study of the physicochemical properties of nanoparticles with different structural characteristics, their interaction characteristics with cancer cells and their abilities as drug carrier systems and thermochemotherapy treatment systems was conducted. Specifically, we aimed to develop new drug carrier systems with the potential of photothermal effect and explore the relationship between their abilities and configurations.

## Materials and methods

DA-HCl, ethanol and ammonia (23–28%) were purchased from Sinopharm (China). P123, F127, 1,3,5-trimethylbenzene (TMB) and cisplatin were bought from Sigma-Aldrich (St Louis, USA). Trypsin, Dulbecco’s modified Eagle’s medium (DMEM) and fetal bovine serum (FBS) were obtained from Gibco (CA, USA). SCC7 cell lines were provided by the State Key Laboratory of Oral Diseases, West China Hospital of Stomatology, Sichuan University (Sichuan, China). Calcein-AM/PI Double Staining Kit was purchased from Solarbio (Beijing, China); Cell Counting Kit-8 (CCK-8) was from Bimake (Houston, TX, USA); Matrigengel was from ABW (Shanghai, China) and Annexin V-FITC/PI Apoptosis kit was obtained from MultiSciences (Hangzhou, China). Female C3H mice (6 weeks old) were purchased from Vital River Laboratory Animal Technology (Beijing, China). They were housed in a specific-pathogen-free (SPF) environment with free access to standard food and water. All animal experiments were approved by the Research Ethics Committee of West China Hospital of Stomatology (No. WCHSIRB-D-2021-561) and were carried out in compliance with the approved guidelines.

### Synthesis of nPDA and nPDA-cis

#### Preparation of three types of nanoparticles

For the preparation of nPDA balls, 250 mg DA-HCl was mixed with 100 ml ultrapure water; 40 ml anhydrous ethanol was added while stirring; and 0.5 ml ammonia was added quickly. The mixture was stirred at 37°C for 8–12 h. The above reaction suspensions were collected and centrifuged at 5–10°C and 13 000 r/min for 10 min. For the preparation of nPDA plates, 200 mg DA-HCl was mixed with 90 ml ultrapure water and stirred at 70°C for 30 h. For the preparation of porous nPDA balls, 5 ml ultrapure water was mixed with 5 ml ethanol, and 25 mg P123, 75 mg F127, 150 mg DA-HCl and 0.4 ml TMB were added. The suspension was thoroughly mixed by ultrasonication for 1 min. Under magnetic stirring, 0.2 ml ammonia was added and incubated at 25°C for 2 h. The above reaction suspensions were collected and centrifuged at 5–10°C and 10 000 r/min for 10 min. The supernatant was discarded, and the pellet was suspended with ultrapure water. Resuspension and centrifugation were repeated 4–5 times until the supernatant was clear and three types of nPDA particles were obtained.

#### Preparation of nPDA-cis particles

According to the different mass ratios of cisplatin with nPDA particles in different experiments, the cisplatin drug was added to the three different configurations of nPDA particle solutions, respectively, and the volume of ultrapure water was replenished to 10 ml so that the final concentration reached the requirements. The reaction was stirred at 37°C at constant temperature in the dark for 24 h to obtain the successful nPDA-cis particles.

The chemical reagents used in the synthetic process are listed in Supplementary Table S1. The different mass ratios of cisplatin to nPDA particles are shown in Supplementary Table S2. More details of the preparation of nPDA and nPDA-cis mentioned above are described in the Supplementary data of this article.

### Material characterization

The morphology, chemical structure and element spatial distribution were determined using scanning electron microscopy (SEM, Thermo Scientific, Waltham, MA, USA), Talos F200S transmission electron microscopy (TEM, Thermo Scientific, Eindhoven, Netherlands) and Fourier-transform infrared spectroscopy (FTIR, Thermo Scientific, Waltham, MA, USA). The prepared nPDA-cis were characterized for their zeta potential (mV) using NanoBrook Series size and zeta potential analyzers (Brookhaven Instruments, New York, USA). To evaluate the temperature change of nPDA and nPDA-cis by the photothermal effect, 1 ml of each sample was added to a 1.5-ml EP tube and irradiated using a near-infrared (NIR) laser source at 808 nm and 0.5 W/cm^2^ for 10 min (Laserwave, Beijing, China). Photographs and temperature were recorded every 40 s by infrared thermal camera (FLIR, Washington, USA). Then, we repeated the 10-min on and 10-min off laser irradiation procedures for three cycles to investigate the stability of the photothermal effect. Photographs and temperature were recorded every 2 min. Temperatures at the upper, middle and lower parts of the suspension were recorded in each photograph.

### Sustained release and controlled release

To investigate the sustained release of nPDA-cis, 10 ml nPDA-cis suspension (2 mg/ml nPDA and 0.8 mg/ml cisplatin) was placed in a dialysis bag, sealed with a clip and placed in a beaker filled with 100 ml PBS buffer (pH 5.8). A total of 10 ml of PBS sample was taken at 0.5, 1, 2, 3, 4, 5, 6, 8, 10, 12, 24, 48, 72, 96, 120 and 168 h, and 10 ml PBS was added each time to maintain the PBS volume at 100 ml. To investigate the photothermal effect on the controlled release of nPDA-cis, NIR laser irradiation (808 nm, 0.5 W/cm^2^, 5 min) was applied to the sample in the dialysis bag, and 10 ml PBS sample was collected afterward. Cisplatin concentration in the sample solution was detected using inductively coupled plasma–optical emission spectroscopy (ICP-OES).

### Cell experiments *in vitro*

In this part, in all of the solutions containing cisplatin, the concentration of cisplatin was 5 μg/ml, and in all of the solutions containing nPDA, the concentration of nPDA was 12.5 μg/ml. The wavelength of the NIR laser was 808 nm, and the irradiation continued for 5 min, on the power of 0.5 W/cm^2^.

#### Cell culture

SCC7 cells were generously provided by State Key Laboratory of Oral Diseases, Sichuan University, and cultured in DMEM supplemented with 10% FBS solution at 37°C in 5% CO_2_ atmosphere.

#### Live/dead assay

The experiment was divided into 16 groups as shown in Table 1, and in each group, we used DMEM with 10% FBS to prepare the media. A total of 5 × 10^4^ cells/well were seeded into 24-well plates and incubated at 37°C in an atmosphere of 5% CO_2_ overnight. Then, the old media were removed, and 1 ml of fresh media with or without nanomaterials or cisplatin was added to each well. Half of the groups were exposed to NIR irradiation (808 nm, 0.5 W/cm^2^, 5 min). Then, all groups of cells were incubated for 6 h. The old media in each well were replaced by 1 ml PBS containing 2 μM Calcein-AM and 4.5 μM PI. The images of cells were taken under an inverted fluorescence microscope (Leica, Wetzlar, Germany) after 20 min of incubation.

**Table 1.** Groups examined by live/dead assay, colony formation assay, scratch wound assay, transwell assays and apoptosis assay

Treatment	DMEM (10% FBS)	Cisplatin	nPDA balls	nPDA plates	Porous nPDA balls	nPDA-cis balls	nPDA-cis plates	Porous nPDA-cis balls
NIR laser	+	+	+	+	+	+	+	+
NIR laser	−	−	−	−	−	−	−	−

#### CCK-8 assay

The experiment was divided into 32 groups as shown in Table 2, using DMEM with 10% FBS to prepare the media. A total of 5 × 10^3^ cells/well were seeded into 96-well plates and incubated at 37°C in an atmosphere of 5% CO_2_ overnight. Then, the old media were removed, and 100 μl of fresh media with or without nanomaterials or cisplatin was added to each well, with each group having six repeat wells. After NIR irradiation (808 nm, 0.5 W/cm^2^, 5 min), the cells were incubated for 24 h or 48 h. Then, we removed the old media and added 100 μl fresh media with 10% CCK-8 reagent to each well, followed by incubation for 1 h at 37°C. After the incubation, the absorbance of each well at 450 nm was measured with a microplate reader. The experiments were repeated three times. The cell inhibition rate was calculated as follows:

(1) Cell inhibition rate (%) = [(Ac-As)/(Ac-Ab)] × 100%

**Table 2.** Groups examined by CCK-8

Condition	DMEM (10% FBS)		Cisplatin		nPDA balls		nPDA plates		Porous nPDA balls		nPDA-cis balls		nPDA-cis plates		Porous nPDA-cis balls	
NIR laser	+/−	+/−	+/−	+/−	+/−	+/−	+/−	+/−	+/−	+/−	+/−	+/−	+/−	+/−	+/−	+/−
Culture time (h)	24	48	24	48	24	48	24	48	24	48	24	48	24	48	24	48

As: experimental well (SCC7 cell + treatment + CCK8); Ac: control well (SCC7 cell + CCK8); Ab: blank well (medium + CCK8).

#### Colony formation assay

To study colony formation, SCC7 cells (200 cells/well) were seeded in six-well plates and incubated overnight. Then, the old media were removed, and new media prepared as shown in Table 1 were added. Half of the groups were treated with NIR irradiation (808 nm, 0.5 W/cm^2^, 5 min). Colony formation was assessed 7 days after the treatment. The colonies were fixed with 4% paraformaldehyde and stained with 0.1% crystal violet.

#### Scratch wound assay

The experiment was divided into 16 groups as shown in Table 1. SCC7 cells (2 × 10^5^ cells/ml, 2.5 ml/well) were seeded in 6-well plates and cultured in DMEM without FBS for 48 h (5% CO_2_, 37°C). After the cells reached 80% confluence, we used 200-μl micropipette tips to create scratch wounds in each well and removed the cell debris by washing with PBS. Then, the media (without FBS) with drugs or nanoparticles prepared as shown in Table 1 were added. After the application of NIR laser (808 nm, 0.5 W/cm^2^, 5 min) in eight groups, the cells were incubated for another 24 h. Cell images for each condition were photographed with an inverted fluorescence microscope at 0 and 24 h. We measured the scratch area with ImageJ software, and compared the scratch area between the beginning and the end of the experiment.

#### Transwell migration and invasion assay

The transwell migration assay and transwell invasion assay were conducted with a Corning transwell chamber. The invasion assay required a layer of Matrigel before cells were added to the chamber, while cell suspension was directly added for the migration assay. The experiment was divided into 16 groups as shown in Table 1. After serum starvation for 48 h, SCC7 cells (5 × 10^4^ cells/well, 100 μl) were seeded in the upper chamber, while 600 μl DMEM (20% FBS) with drugs or nanoparticles prepared as in Table 1 was added to the lower chamber. Half of the groups were exposed to NIR laser (808 nm, 0.5 W/cm^2^, 5 min). A cotton swab was used to scrub the remaining cells in the upper chamber after cultivation for 24 h. The cells that migrated/invaded through the chamber membrane were fixed and washed, and stained with 0.1% crystal violet for 20 min. The migrated cells were counted in five images per membrane under a 400× objective.

#### Annexin V-FITC and PI dual staining assay

The experiment was divided into 16 groups as shown in Table 1, and the media with or without drugs and nanoparticles were prepared using DMEM with 10% FBS. After 12 h of incubation, the cells were trypsinized and washed with PBS twice, and then suspended in 500 μl binding buffer. Then, the cells were stained with 5 μl Annexin V-FITC and 10 μl PI and incubated at room temperature for 5 min in the dark. The stained cells were analyzed by flow cytometer of Beckman Coulter (California, USA), and the data were analyzed using FlowJo X10 (Ashland, USA).

### *In vivo* experiments: mouse homograft model

Sixty 6-week-old female C3H mice (Charles River, Beijing, China) weighing 20 ± 2 g were housed at 21°C on a 12 h light/12 h dark cycle with free access to food and water, and bedding was changed twice a week. SCC7 cells were digested with 0.25% trypsin and centrifuged at 800 r/min for 5 min. Cell pellet was collected and washed twice with PBS and resuspended for cell counting. Cell density was adjusted to 1 × 10^7^/ml with PBS and kept on ice until use. The mice were anesthetized by intraperitoneal injection of 0.3% sodium pentobarbital solution (0.1 ml/10 g). They were marked with ear tags and weighed. The axilla of the right forelimb was shaved; the skin was disinfected with ethanol; and the cell suspension was injected at a dose of 100 μl/mouse. Subcutaneous tumor appeared on Day 5 after injection. The mice were weighed and grouped according to Table 3, with six mice in each group. There were no significant differences in body weight between the groups.

**Table 3.** Groups examined in a mouse homograft model

Treatment	Normal saline (NS)	Cisplatin	nPDA-cis balls	nPDA-cis plates	Porous nPDA-cis balls
NIR laser	+	+	+	+	+
NIR laser	−	−	−	−	−

Three nPDA-cis injections were prepared with normal saline. The final concentration of cisplatin was 0.8 mg/ml, and the final concentration of nPDA was 2 mg/ml. The mice were anesthetized by intraperitoneal injection of 0.3% sodium pentobarbital solution (0.1 ml/10 g) on Day 1. Cisplatin, nPDA-cis, or normal saline were injected into the tail vein at 200 μl/mouse. Half of the mice in each group were locally irradiated with an NIR laser (808 nm, 0.5 W/cm^2^, 5 min) at 6 h after injection, and body weight was measured every 3 days thereafter. The longest diameter (*a*) and the shortest diameter (*b*) of the tumor were measured using a vernier caliper, and tumor volume was calculated using the following equation:

(2) $$V\;=\;\frac{1}{2}\times a\times b^{2}$$

A tumor volume of 2000 mm^3^ was chosen as a humane endpoint, and the mice were sacrificed by sodium pentobarbital overdose.

### Immunohistochemical staining and scoring

The mice were sacrificed for tumor dissection. Each tumor was embedded in paraffin and sectioned, followed by immunohistochemical staining for Ki-67, Caspase-3, *γ*-H2AX and MMP-9. Ten 400× fields were randomly selected from each section for scoring under microscope (Leica). The scoring criteria were as follows: 1 (<25% positive cells); 2 (25–50% positive cells); 3 (50–75% positive cells); and 4 (> 75% positive cells). The staining intensity scoring criteria were as follows: 0 = no staining, 1 = light brown, 2 = brown, and 3 = dark brown. The two scores were multiplied to obtain a final immunohistochemical score: 0, negative (−); less than 4, weakly positive (+); 4–8, moderately positive (++); and greater than 8, strongly positive (+++).

### Statistical analysis

Software SPSS (SPSS version 20, SPSS Inc., Chicago, IL, USA) was used for the experimental data analysis, and software GraphPad Prism (GraphPad Prism 8.1.2, GraphPad Software, San Diego, California, USA) was used for plotting. Differences between groups were determined using the *t-*test. Student’s *t-*test was chosen when the two groups of samples have equal variances. If samples have unequal variances, Welch’s *t-*test was used. Statistical significance was defined as *P < 0.05, **P < 0.01 and ***P < 0.001. Data were presented as mean ± SD.

## Results

### Basic properties of different configurations of nPDA and nPDA-cis

**Figure 1.**
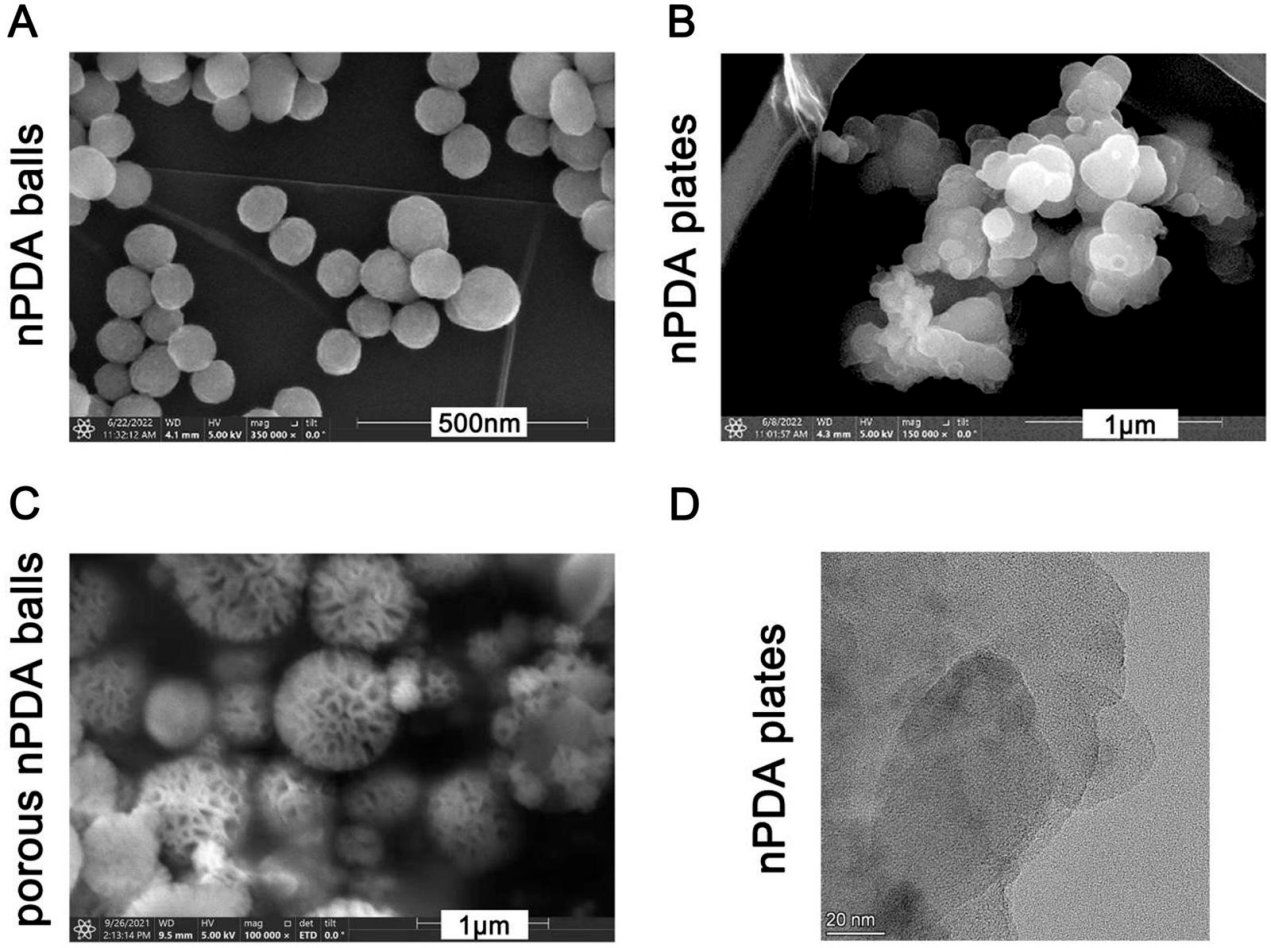
Spatial properties of different configurations of the nPDA. (**A**) SEM imaging of nPDA balls. (**B**) SEM imaging of nPDA plates. (**C**) SEM imaging of porous nPDA balls. (**D**) TEM imaging of nPDA plates.

**Figure 2.**
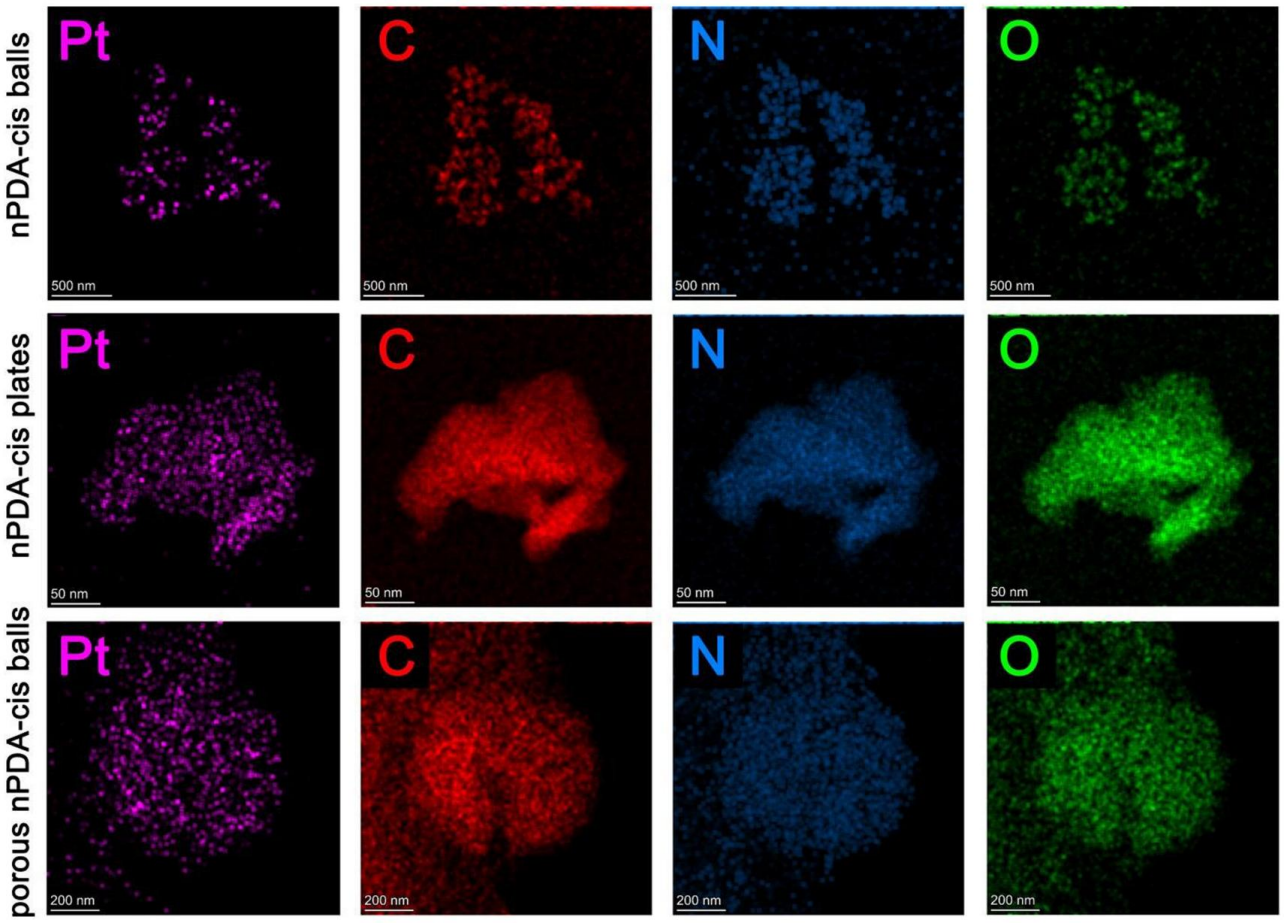
Element distribution images of nPDA-cis by HAADF-STEM. The letter in the upper left corner of the image is the name of the chemical element shown in the image.

Firstly, we evaluated the synthesized nPDA via SEM and TEM (Figure 1), and we evaluated nPDA-cis via HAADF-STEM (Figure 2). Among the three different configurations of nPDA particles, the nPDA balls had the smallest size and uniform morphology, with the vast majority of them having the particle size of about 90 nm; the nPDA plates had irregular morphology and the longest diameter above 1 μm, and the surface was rough; the porous nPDA balls showed a spherical, porous and sparse structure with prominent size variations, where the largest porous nanospheres reached 800 nm in diameter, while the smallest porous nanospheres had a diameter of about 100 nm (Figure 1). After loading cisplatin onto these three configurations of nPDA particles, we obtained the element distribution images of nPDA-cis by HAADF-STEM, according to the HAADF-STEM results, the element Pt was observed inside all three different configurations of nPDA-cis, and its spatial distribution was evenly dispersed (Figure 2). These results indicated that nPDA-cis was successfully constructed, and all three configurations of nPDA particles were different in terms of morphology.

**Figure 3.**
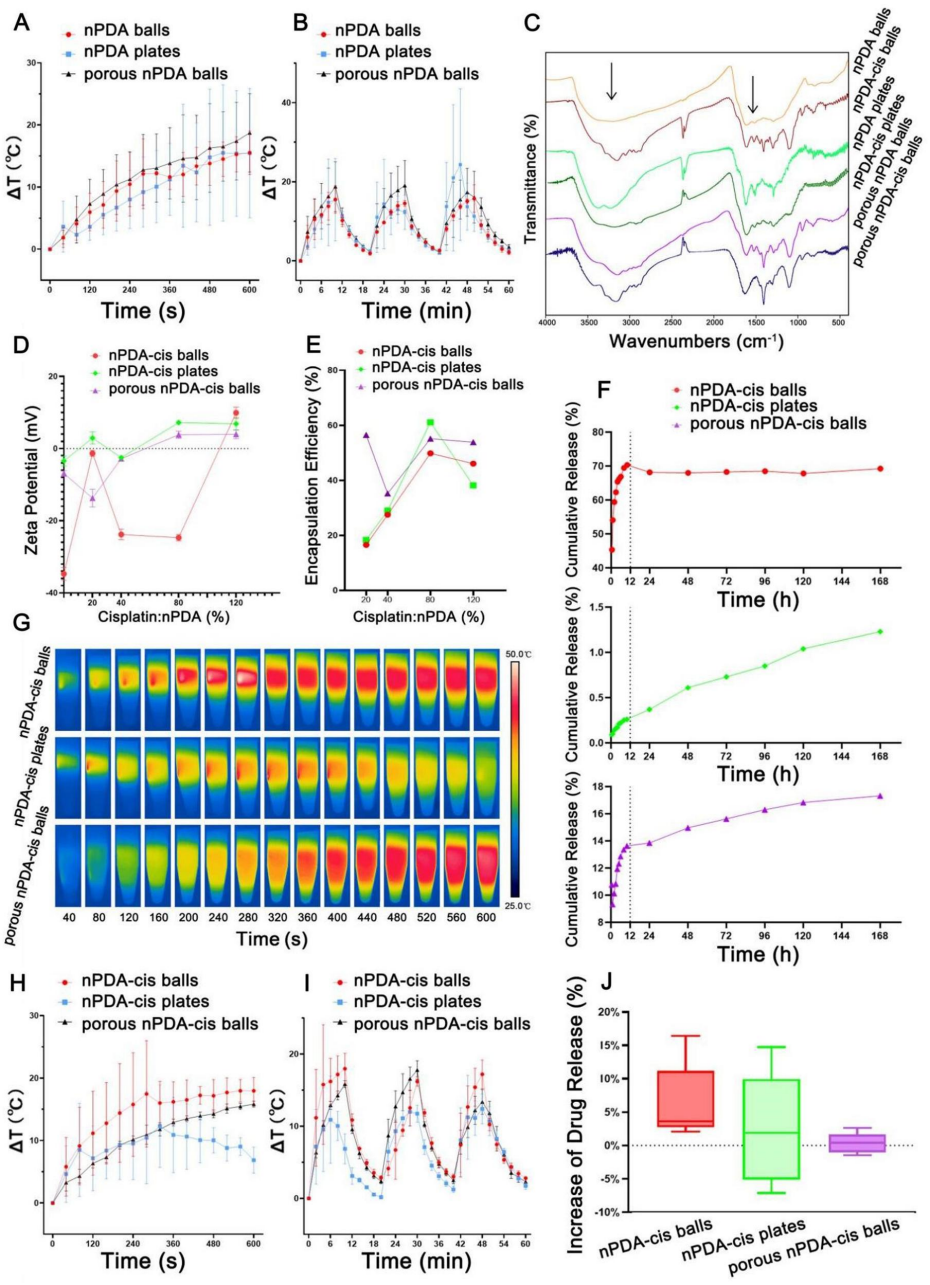
Photothermal and drug-loading properties of the different configurations of nPDA and nPDA-cis. (**A**) Photothermal temperature curves of nPDA. (**B**) The temperature changes of the nPDA over three lasers on/off cycles (808 nm, 0.5 W/cm^2^). (**C**) FTIR. (**D**) Zeta potential. (**E**) Encapsulation efficiency. (**F**) Cumulative release. (**G**) Infrared thermal images of nPDA-cis. (**H**) Photothermal temperature curves. (**I**) The temperature changes of the nPDA-cis over three lasers on/off cycles (808 nm, 0.5 W/cm^2^). (**J**) Increased rate of cumulative release.

Then, we evaluated the photothermal and drug-loading properties of the three different configurations of nPDA and of nPDA-cis (Figure 3). The three configurations of nPDA exhibited various photothermal properties (Figure 3A and B). During the 10-min NIR laser application and temperature rise process, a low power and short exposure time were sufficient for all three to achieve the required temperature for clinical treatment (Figure 3A). But it is noteworthy that among these configurations, the rate of temperature rise and the uniformity of temperature change in space differed, namely, the temperature rise was at a more uniform rate with the nPDA balls and porous nPDA balls, and at a slightly less uniform rate with the nPDA plates (Figure 3A). In addition, in the three cycles of photothermal effect of temperature rise and fall, the nPDA balls particles repeatedly showed the most stable temperature rise performance in multiple cycles, and the temperature rise was uniform at all points in the liquid, while the nPDA plates had a highly unstable temperature rise rate in the cycle, and the internal temperature of the liquid appeared to rise significantly and unevenly (Figure 3B).

As shown in Figure 3C, the three different configurations of nPDA and the three different configurations of nPDA-cis have different functional group characteristics. In the characteristic frequency region of 4000–1330 cm^−1^, they all exhibited the functional group features of PDA, including the N–H characteristic bond in the range of 3500–3100 cm^−1^, and the characteristic peaks of the indole structure formed from the self-assembly of DA-HCl monomer were exhibited in the range of 1600–1500 cm^−1^, where multiple characteristic peaks also represent the presence of aromatic rings. In the fingerprint region of 1330–400 cm^−1^, the three different configurations of nPDA and nPDA-cis exhibited different characteristic peaks for the drug-loaded system (Figure 3C). Overall, the nPDA-cis curve was more complex and fluctuated more significantly, which might be due to the effect of cisplatin on its structure (Figure 3C). Since PDA is insoluble in water and most organic solvents, its exact composition and structure remain controversial [27]. The generally accepted view is that at the very initial stage of the reaction, dopamine autoxidizes and forms dopamine-quinone, followed by 5,6-dihydroxyindole (DHI), but the subsequent re-formation of PDA is very complex, and the exact pathway is still inconclusive [28].

Through the Zeta potential of nPDA-cis at different cisplatin/nPDA ratios, we found that the surface charge of nPDA particles was negative, and the surface charge of nPDA-cis particles changed with increasing mass of cisplatin in the system (Figure 3D). The surface charge of nPDA-cis plates began to change from negative to positive at a mass ratio of ∼50%, and that of porous nPDA-cis balls began to change at ∼60% (Figure 3D). When the mass ratio reached 80%, the surface charge of nPDA-cis plates and porous nPDA-cis balls fully changed to positive (Figure 3D). The surface charge of nPDA-cis balls began to change from negative to positive at ∼110%, and the positivity continued to increase at 120% (Figure 3D).

The results of the encapsulation efficiency of the three different configurations of nPDA-cis after 24 h of reaction revealed that the encapsulation efficiency was not simply linearly related to the cisplatin/nPDA ratio (Figure 3E). The encapsulation efficiency of nPDA-cis balls increased with the increase of cisplatin/nPDA mass ratio, and was basically stable after the mass ratio of the injected cisplatin to nPDA reached 80% (Figure 3E). Similarly, the encapsulation efficiency of nPDA plates also saturated at the mass ratio of 80% and showed little decrease with further increase of cisplatin (Figure 3E). It is worth noting that the encapsulation efficiency of porous nPDA balls was special compared with the other two configurations of nPDA. The encapsulation efficiency of porous nPDA-cis balls decreased when the mass ratio increased from 20% to 40% (with drug content remaining stable) and then increased at >40%, which may be related to the change of the surface charge (from negative to positive) around this mass ratio (Figure 3E).

The one-week cumulative release curves showed that the final release of nPDA-cis balls reached about 70% (Figure 3F). nPDA-cis plates had a more stable rate of release increase throughout the 1-week period, but the final release was extremely low, less than 1.5% at Day 7 (Figure 3F). The release pattern of porous nPDA-cis balls was somewhere in between, being the fastest within the initial 12 h, reaching 14% at 12 h, and gradually slowing down between 12 h and 168 h (Day 7), with its final release approaching 18% (Figure 3F). Although the trend was consistent with slow release, the final release was less than ideal. These results may be related to the excess reactive groups on the surface of plates and porous balls, which formed a strong chelation with Pt [29, 30].

In terms of the heating rate and spatial uniformity of the three different configurations of nPDA-cis, nPDA-cis balls and porous nPDA-cis balls exhibited good heating performance, in contrast to nPDA-cis plates, which showed unstable and nonuniform heating and small temperature increase (Figure 3G and H). These results indicate the influence of the configuration on the physical properties. Although the temperature increase of the three different configurations of nPDA-cis could still meet the clinical requirements after carrying cisplatin, the differences in heating performance became larger (Figure 3H). Specifically, the heating capacity of nPDA-cis balls remained the strongest, but the overall heating capacity of nPDA-cis plates decreased significantly (Figure 3H).

The temperature changes of the nPDA-cis over three laser on/off cycles show that the nPDA-cis balls had a relatively uniform heating over the three cycles; porous nPDA-cis balls had a more spatially uniform heating, but the temperature increase varied over the three cycles, and nPDA-cis plates had the lowest temperature increase and the largest internal temperature difference spatially (Figure 3I). These results suggest that the configuration has a great impact on the photothermal performance of interest in practical clinical applications.

In order to investigate the effect of NIR laser irradiation on the controlled release of different configurations of nPDA-cis, we conducted an experiment to detect the release degree again after 5 min of NIR laser irradiation, and the results are shown in Figure 3J. Among the three configurations, nPDA-cis balls showed the largest change in drug release after light irradiation, and the effect of drug release increased most stably in multiple experiments, up to more than 16% (Figure 3J). In contrast, porous nPDA-cis balls showed almost no effect of photothermal controlled release (Figure 3J). For nPDA-cis plates, although the highest degree of release variation was close to 15%, its photothermal controlled release effect was extremely unstable due to its small release base (Figure 3J). Overall, the nPDA-cis balls have the most optimal controlled release effect.

In conclusion, nPDA-cis balls and porous nPDA-cis balls demonstrate better uniformity and stability in temperature rise during NIR laser irradiation compared to nPDA-cis plates, which show less uniform and unstable temperature profiles. This implies that nPDA-cis balls and porous nPDA-cis balls could provide more effective and predictable photothermal therapy.

### Efficacy of different configurations of nPDA and nPDA-cis *in vitro*

**Figure 4.**
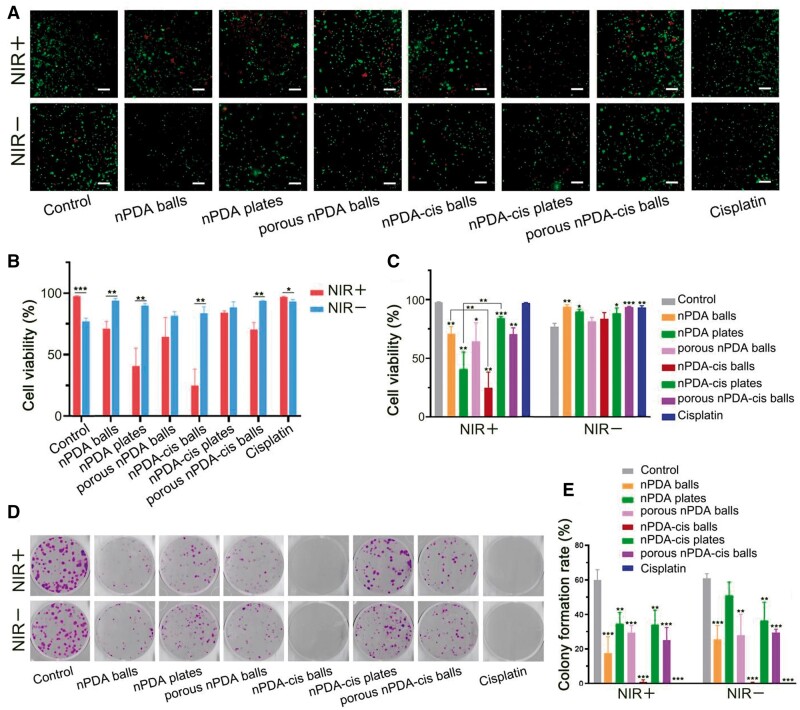
Live/dead cell staining and colony formation assay by different configurations of nPDA and nPDA-cis. The asterisks above the column show the statistical significance of the comparison between each group and the blank treatment group under the same incubation time and NIR laser exposure (*P < 0.05, **P < 0.01, ***P < 0.001). (**A**) Live/dead cell staining, scale label = 200 μm. The red cells are dead cells and the green ones are live cells, the statistical results are shown in (**B**) and (**C**). (**D**) Results of colony formation assay. (**E**) Colony formation rate.

**Figure 5.**
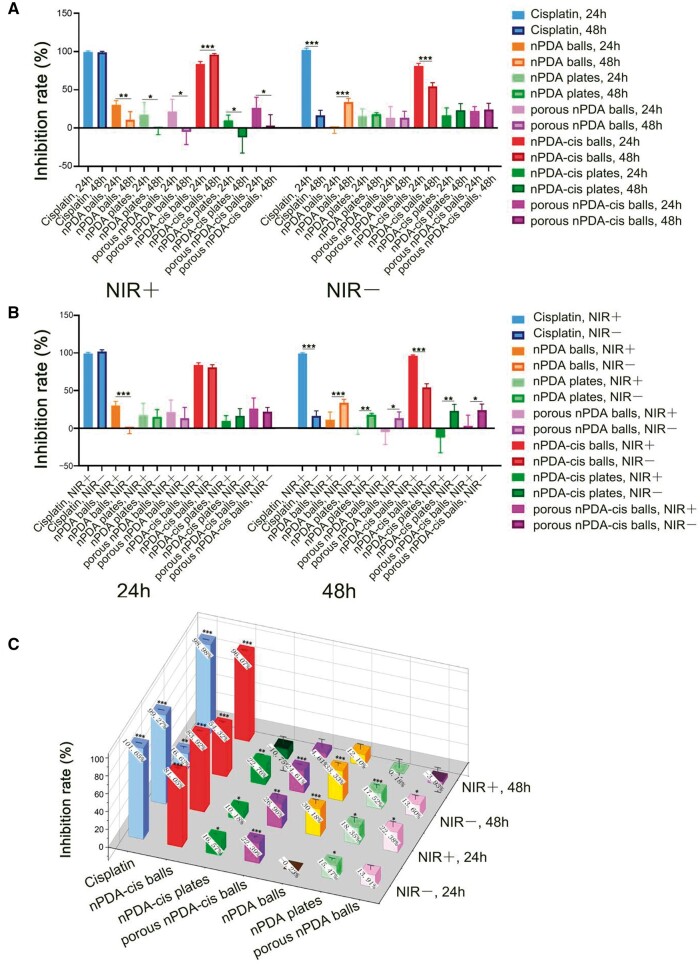
Inhibition of tumor cell proliferation *in vitro* by different configurations of nPDA and nPDA-cis. The asterisks above the column show the statistical significance of the comparison between each group and the blank treatment group under the same incubation time and NIR laser exposure (*P < 0.05, **P < 0.01, ***P < 0.001). (**A**) Inhibition rate obtained using the Cell Counting Kit-8 with or without NIR laser. (**B**) Inhibition rate obtained using the CCK-8 at 24 and 48 h. (**C**) The inhibition rate was obtained using the CCK-8 under different treatments.

**Figure 6.**
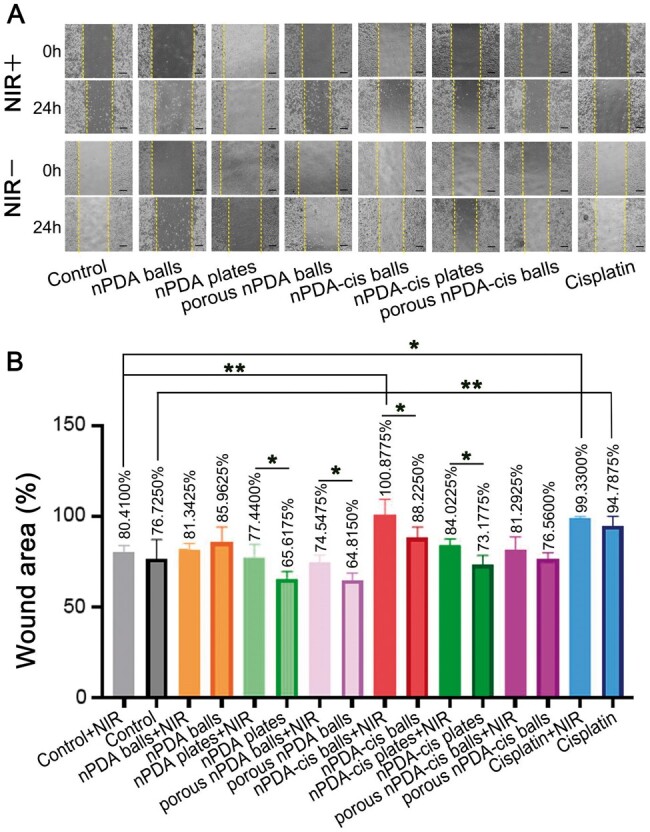
Tumor cell migration induction by different configurations of nPDA and nPDA-cis (*P < 0.05, **P < 0.01, ***P < 0.001). (**A**) Scratch wound assay, scale label = 200 μm. (**B**) Change of wound area (the ratio of the final area to the initial area).

**Figure 7.**
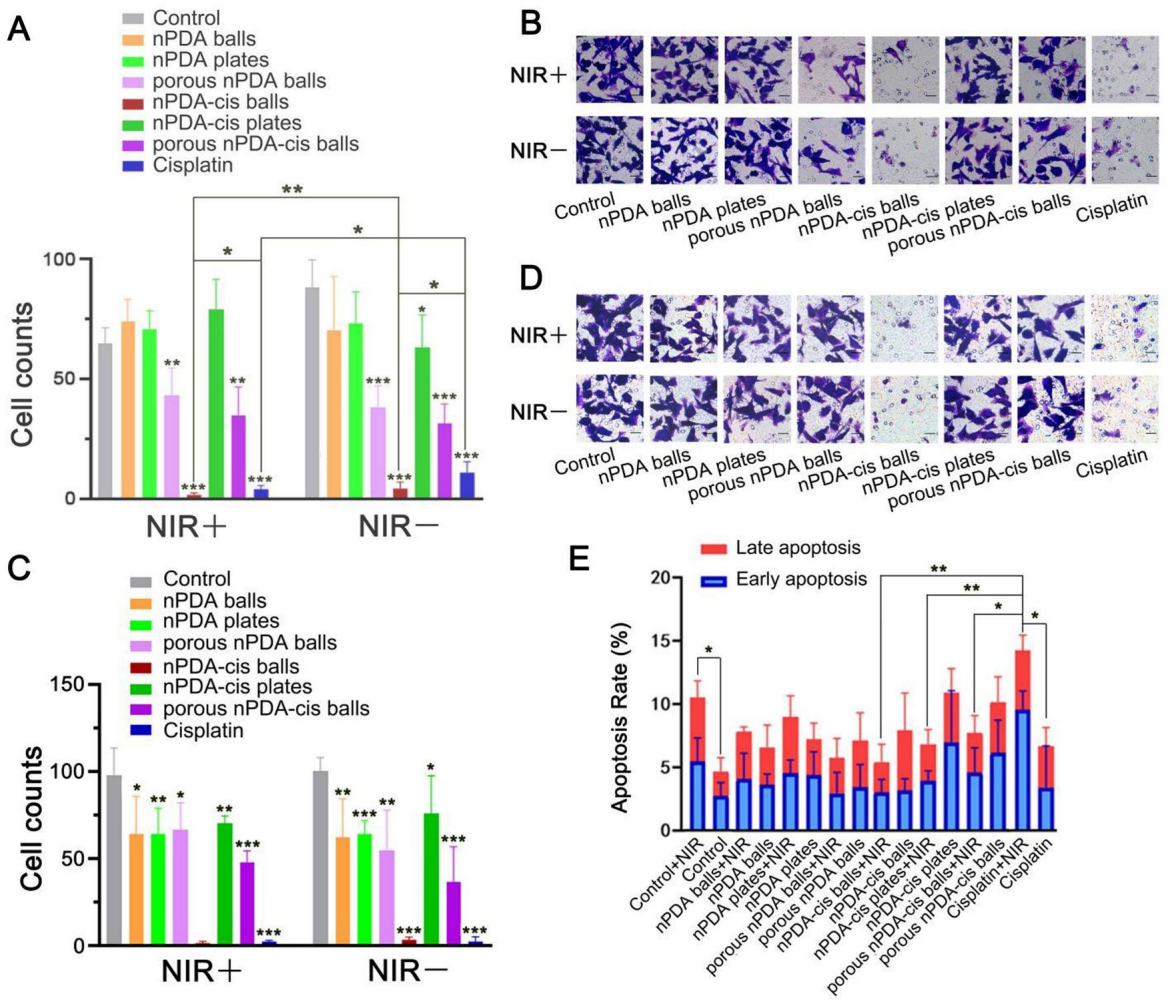
Tumor cell migration, invasion and apoptosis induction by different configurations of nPDA and nPDA-cis. The asterisks above the column show the statistical significance of the comparison between each group and the blank treatment group under the same incubation time and NIR laser exposure (*P < 0.05, **P < 0.01, ***P < 0.001). (**A**, **B**) Transwell migration assay, the cells migrated through the chamber, scale label = 50 μm. (**C**, **D**) Transwell invasion assay, the cells invaded through the chamber, scale label = 50 μm. (**E**) Apoptosis rate.

After characterizing the physical and chemical properties, we evaluated the effects of three different configurations of nPDA and three different configurations of nPDA-cis drug carrier systems on the biological behavior of tumor cells *in vitro* (Figures 4–7). The results of live-dead staining are shown in Figure 4A–C, where green represents live cells and red represents dead cells (Figure 4A). The cell viability charts show that the survival rate of the three configurations of nPDA, after 6 h of incubation, is not lower than that of the blank group (Figure 4B and C), and it can be assumed that these three kinds of nanoparticles are not acutely cytotoxic.

The cell survival rate with photothermal treatment was lower in all different configuration nPDA and nPDA-cis groups, with statistically significant differences (*P* < 0.01) within the nPDA balls, nPDA plates, nPDA-cis balls and porous nPDA-cis balls groups (Figure 4B). This indicates that the temperature increase caused by the photothermal effect in each group had a very clear effect on the killing of SCC7 cells in a short period of time. However, the survival rate difference between the experimental groups of nPDA-cis plates with and without photothermal treatment was extremely small and not statistically significant, probably due to the low-temperature increase of nPDA-cis plates after NIR laser irradiation (Figure 3H), which was not able to effectively kill SCC7 cells in a short period of time.

In the groups with photothermal treatment, the cytotoxicity of nPDA-cis balls was stronger than that of nPDA balls, while for nPDA-cis plates, the cytotoxicity was weaker than that of nPDA plates (Figure 4C). The above results correspond to the different magnitude of the temperature increase of the photothermal effect for various configurations of nPDA and nPDA-cis (Figure 3A and H), which further indicates that it is the photothermal effect that causes the difference in the short-term cell-killing ability. It also indicates that nPDA and cisplatin produced synergistic effects under photothermal induction.

The colony formation assay revealed that the cisplatin group and nPDA-cis balls group (with or without NIR irradiation) had the best ability to inhibit tumor cell proliferation (Figure 4D), and there was no statistically significant difference in the colony formation rate between the two groups (Figure 4E).

Among the configurations of nPDA and nPDA-cis, all groups had different degrees of inhibitory effects on tumor cell proliferation, but the nPDA plates without NIR laser treatment were not significantly different from the blank group (Figure 4E). For the same configuration of nanoparticles, the clone formation rates had no statistically differences with or without photothermal treatment (Figure 4E). Therefore, we believe that the properties of the nPDA or nPDA-cis system itself, rather than the photothermal effect, are the main factors affecting the proliferative capacity of tumor cells at longer time scales.

Figure 5A shows the inhibition rate at different time points with or without NIR irradiation. For all the experimental groups that received NIR laser irradiation, the inhibition rate of cisplatin showed no statistically significant difference under 24 or 48 h incubation (Figure 5A). However, the inhibition rate of all three configurations of nPDA particles, the nPDA-cis plates and porous nPDA-cis balls, decreased with time, indicating that the inhibition ability of these groups was mainly from the photothermal effect at the beginning of the experiment (Figure 5A). Notably, for nPDA-cis balls that received NIR laser irradiation, the ability to inhibit cancer cell proliferation was enhanced over time (*P* < 0.001) (Figure 5A and C).

For each experimental group that did not receive NIR laser irradiation, the inhibitory ability of cisplatin and nPDA-cis balls on tumor cell proliferation decreased over time (*P* < 0.001) (Figure 5A). For each of the nPDA and nPDA-cis groups, the inhibition rate in combination with photothermal treatment showed opposite trends in time compared with the group without photothermal treatment (Figure 5A), suggesting that the photothermal effect is the main factor affecting the efficacy in the shorter treatment time. Combination with photothermal treatment or lack thereof resulted in opposite trends in the inhibition rates, suggesting a good chemotherapeutic drug-sensitizing effect of the photothermal effect of nPDA-cis balls.

Figure 5B demonstrates the inhibition rate of each experimental group with different NIR irradiation exposure at different time points. After 24 h of incubation, the ability of different configurations of nPDA or nPDA-cis to inhibit the proliferation of SCC7 cells was almost independent of the photothermal effect, except in the configuration of nPDA balls, where NIR laser irradiation was able to significantly enhance its ability to inhibit SCC7 cell proliferation (Figure 5B). But when the time was extended to 48 h, the effect of photothermal effect on the ability to inhibit the proliferation of SCC7 cells became more significant in all the groups (Figure 5B). For the cisplatin group and nPDA-cis balls, the photothermal effect synergized with the chemotherapeutic effect and significantly enhanced the ability to inhibit tumor cell proliferation (*P* < 0.001) (Figure 5B). In contrast, for nPDA plates, porous nPDA balls and nPDA-cis plates, the photothermal effect reduced the ability to inhibit tumor cell proliferation (Figure 5B), suggesting that the photothermal effect may have led to the tolerance of tumor cells to adverse stimuli, which promoted the proliferation of SCC7 cancer cells.

Under the condition of synergy with photothermal treatment, nPDA-cis balls achieved an inhibition rate equivalent to that of cisplatin, and the inhibition rate of nPDA-cis balls without combined photothermal treatment after 48 h was 2.3 times higher than that of the cisplatin-only group (*P* < 0.001) (Figure 5C), indicating that this nPDA-cis balls drug carrier system has a good photothermal and chemosensitizing effect. At multiple time points, the inhibition effect of nPDA-cis balls group was superior or equivalent to that of cisplatin, which indicates good tumor treatment effect.

We studied the tumor cell migration induction by different configurations of nPDA and nPDA-cis using scratch wound assay and transwell migration assay (Figures 6 and 7A and 7B). In the scratch wound assay, nPDA-cis plates and porous nPDA-cis balls and all three configurations of nPDA failed to inhibit tumor cell migration (Figure 6A and B). With NIR irradiation, only the cisplatin group and the nPDA-cis balls group significantly inhibited cell migration (*P* < 0.01), and the two groups were statistically equivalent in their inhibitory ability. In the group without photothermal cotreatment, cisplatin inhibited migration, while the nPDA-cis balls group did not inhibit migration (Figure 6B). A comparison with and without photothermal cotreatment revealed that the photothermal effect mostly acted as an inhibitor of cancer cell migration in all groups, but the effect was statistically significant (*P* < 0.05) only in nPDA plates, porous nPDA balls, nPDA-cis balls and nPDA-cis plates (Figure 6B).

Similar results were obtained in the transwell experiments (Figure 7A and B). The inhibition of SCC7 cancer cells migration in the cisplatin and nPDA-cis balls groups was highly significant (*P* < 0.001) (Figure 7A). The inhibition of migration was enhanced in both groups in concert with photothermal treatment, while the increase was more significant in nPDA-cis balls group (Figure 7A). The inhibition of migration by nPDA-cis balls was higher than that by cisplatin alone with or without photothermal treatment (*P* < 0.05) (Figure 7A). In the other groups, porous nPDA balls and nPDA-cis had some ability to inhibit migration of SCC7 cancer cells, while the plate configuration did not significantly inhibit migration (Figure 7A).

The different configurations of nPDA and nPDA-cis also had effect on the invasion ability of cancer cells (Figure 7C and D). Transwell invasion assay showed that all experimental groups were able to inhibit the invasion ability of SCC7 cells to varying degrees, but the combination with photothermal treatment versus the lack thereof had no obvious difference in the ability of tumor cells to invade. Therefore, the influence of the photothermal effect on invasion ability was not obvious (Figure 7C and D).

The effect of the cisplatin group, nPDA-cis balls group and porous nPDA-cis balls group to inhibit invasion was very significant (*P* < 0.001) (Figure 7C). Among them, the inhibition effect was stronger in the first two groups, and the ability of the nPDA-cis balls group to inhibit cancer cell invasion was statistically equivalent to that of the cisplatin group (Figure 7C). The nPDA-cis plates had some ability to inhibit invasion, but it was weaker than that of the porous nPDA-cis balls group (*P* < 0.05) (Figure 7C). As for the non-drug-loaded nPDA particles of the three configurations, their ability to inhibit tumor cell invasion was relatively similar, and ANOVA revealed no statistically significant difference between the six experimental groups with or without the use of NIR laser irradiation (Figure 7C).

The above results again suggest that nPDA-cis balls may be a drug carrier system with excellent application prospects.

In general, photothermal treatment could synergize with chemotherapy to promote the inhibition of cell migration in the most efficacious groups, which were cisplatin and nPDA-cis balls. But the effect of photothermal treatment on migration was unclear for the other groups. nPDA-cis balls had the strongest migration inhibition ability and were superior or equivalent to cisplatin in combination with or without photothermal treatment (Figure 7A).

The effect of different configurations of nPDA on invasion ability had no obvious difference. The effect of nPDA-cis on the invasion ability of tumor cells was mainly related to the amount of cisplatin released. The effect of photothermal treatment on invasion ability is not clear (Figure 7C).

The results of the apoptosis assessment (Figure 7E) showed that NIR irradiation had different effects on different groups; specifically, NIR irradiation increased the rate of apoptosis in a short period of time in cisplatin and blank control (*P* < 0.05). In addition, for various configurations of nPDA or nPDA-cis groups, the presence or absence of NIR irradiation did not produce a significant effect on the apoptosis rate (*P* > 0.05) (Figure 7E). Comparing the different groups, we found that the apoptosis rate in all of the nPDA-cis groups combined with photothermal treatment was significantly lower than that in the cisplatin group combined with photothermal treatment (*P* < 0.05), while the apoptosis rate in all of the nPDA-cis groups without photothermal treatment was not significantly different from that in the cisplatin group without photothermal treatment (*P* > 0.05) (Figure 7E). This suggests that the photothermal effect may promote the tolerance of cells to adverse stimuli in a short period of time and have the effect of inhibiting apoptosis.

### Efficacy of three different configurations of nPDA-cis *in vivo*

Hyperthermia has been reported to enhance the efficacy of chemoradiotherapy mainly by activating the immune system [6]. In addition, hyperthermia may synergize with cisplatin to reduce drug resistance in cancer cells and survival and thereby enhance efficacy [10]. Drug encapsulation, modification and transport by nanomaterials reduce the off-target toxicity of chemotherapeutic drugs and increase cisplatin accumulation and retention in tissues [14]. Therefore, to truly and effectively illustrate the antitumor effects of nPDA-cis of different structures with or without photothermal treatment, C3H mice with normal immune systems were used to establish an allograft tumor model.

**Figure 8.**
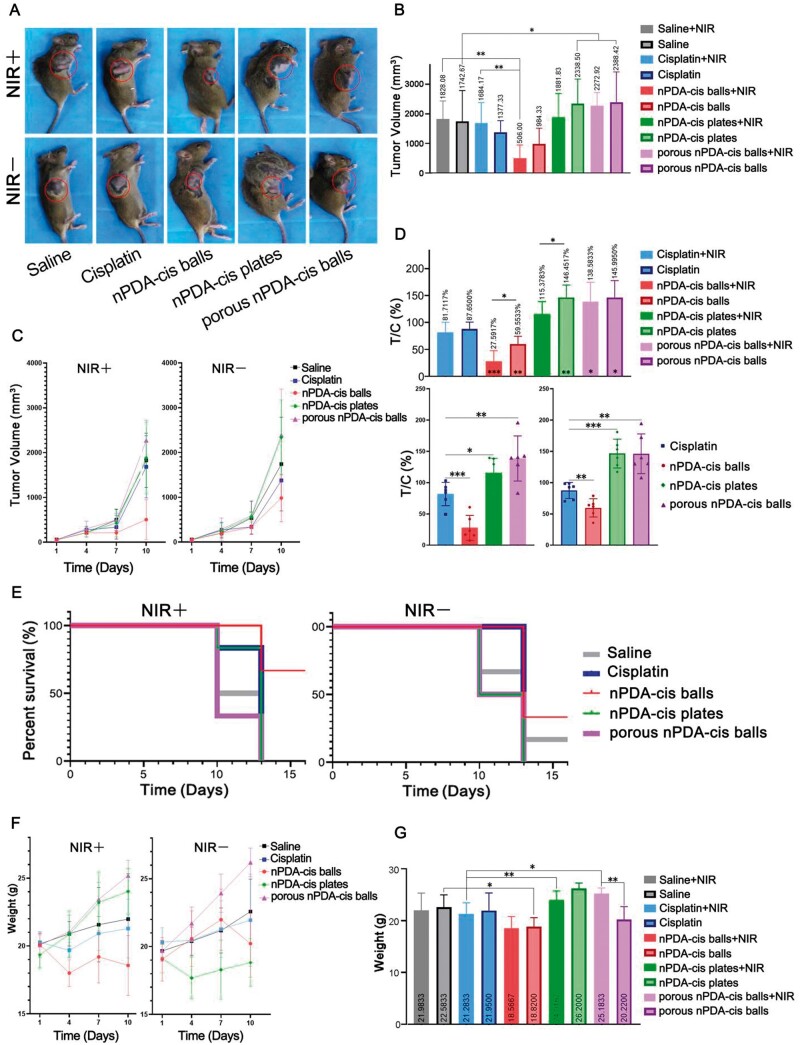
Anti-tumor effects of different configurations of nPDA-cis *in vivo*. The asterisks in the column show the statistical significance of the comparison between each group and the blank treatment group under the same incubation time and NIR laser exposure (*P < 0.05, **P < 0.01, ***P < 0.001). (**A**) Mouse homograft model of SCC7. (**B**) Tumor volume on Day 10. (**C**) Change in tumor volume over time. (**D**) Relative tumor proliferation rate *T*/*C* (%). (**E**) Survival curve. (**F**) Change in weight over time. (**G**) Weight of mice on Day 10.

The model was successfully established 5 days after SCC7 cell injection, and that day was recorded as Day 1 of the tumor-bearing experiment. After 10 days of observation, the tumor volume of some mice reached the humane endpoint (Figure 8A). The tumor volume at the end of the experiment was recorded (Figure 8B), and the tumor volume change over time was also recorded (Figure 8C). On Day 10, the average tumor volume of nPDA-cis balls with the NIR group was significantly smaller than cisplatin with NIR group (*P* < 0.01), and was only 30.04% that of cisplatin with NIR group. Besides, the average tumor volume of nPDA-cis balls group was only 71.47% that of cisplatin group. Apparently, nPDA-cis balls showed the best tumor inhibition with and without NIR laser irradiation, and they significantly outperformed cisplatin.

For mice treated with nPDA-cis plates and porous nPDA-cis balls, tumor volumes were larger than those in the saline control group at the end of the experiment (Figure 8B). Among them, the groups without NIR irradiation were significantly larger than those in the saline group (*P* < 0.05) (Figure 8B). These results suggest that these two configurations of drug carrier systems promoted tumor growth (Figure 8B and C).

To evaluate the therapeutic effects of the three types of nPDA-cis, we performed the following calculations, and the data are plotted in Figure 8D. First, relative tumor volume (RTV) was calculated with the following equation:

(3) $$\mathrm{RTV}\;=\;\frac{V_{t}}{V_{0}}$$

where *V_t_* is the tumor volume measured on day *t*, and *V*_0_ is the tumor volume on Day 1.

The antitumor activity was evaluated using the relative tumor proliferation rate T/C (%), which was calculated using the equation

(4) TC =  TRTVCRTV

where *T*_RTV_ is the RTV of the treatment group and *C*_RTV_ is the RTV of the negative control group (normal saline). *T*/*C* (%) > 40% was considered ineffective, while *T*/*C* (%) ≤ 40% (*P* < 0.05) was considered effective. As a result, nPDA-cis balls with photothermal treatment showed the best therapeutic effect, the relative tumor proliferation rate *T*/*C* was 27.59%, and was statistically significantly better than cisplatin with the photothermal treatment group (*P* < 0.001) (Figure 8D). nPDA-cis balls without photothermal treatment also showed a statistically significantly better therapeutic effect than the cisplatin group (*P* < 0.01) (Figure 8D). nPDA-cis plates promoted tumor growth without photothermal treatment (*P* < 0.01), and porous nPDA-cis balls promoted tumor growth both with and without photothermal treatment (*P* < 0.05) (Figure 8D).

This experiment revealed that the synergistic effect of photothermal treatment combined with chemotherapy could lead to significant differences in antitumor activity. Photothermal treatment significantly reduced tumor proliferation and increased the antitumor activity of nPDA-cis balls and plates (*P* < 0.05) (Figure 8D). For cisplatin and porous nPDA-cis balls groups, the groups with photothermal treatment may have a lower tumor proliferation rate, but there was no statistical significance (Figure 8D). nPDA-cis balls outperformed cisplatin (*P* < 0.01), and the difference was highly significant with photothermal treatment (*P* < 0.001) (Figure 8D).

A mouse was considered dead when the subcutaneous tumor volume reached 2000 mm^3^; the survival curves were plotted (Figure 8E). The survival rate was the highest in the group treated with nPDA-cis balls (Figure 8E), and the value was significantly different between the group treated with nPDA-cis balls with NIR irradiation and the group treated with normal saline (*P* < 0.01) (Figure 8E).

The changes in mouse body weight can reflect the safety of drugs. According to the body weights at the end of the tumor-bearing experiment (Day 10) (Figure 8G), and the change of body weight (Figure 8F), animals exposed to nPDA-cis balls with photothermal treatment, which showed the best therapeutic effect, demonstrated similar body weights relative to the saline control, so nPDA-cis balls can be considered a safe drug carrier system. As shown in Figure 8G, mice exposed to porous nPDA-cis balls demonstrated a significant difference in body weight depending on whether combined with photothermal treatment (*P* < 0.01), which may be related to cachexia caused by large tumors. The differences in body weights in animals exposed to porous nPDA-cis balls and nPDA-cis plates relative to those exposed to cisplatin may be explained by the relatively small tumor volume in the cisplatin group.

**Figure 9.**
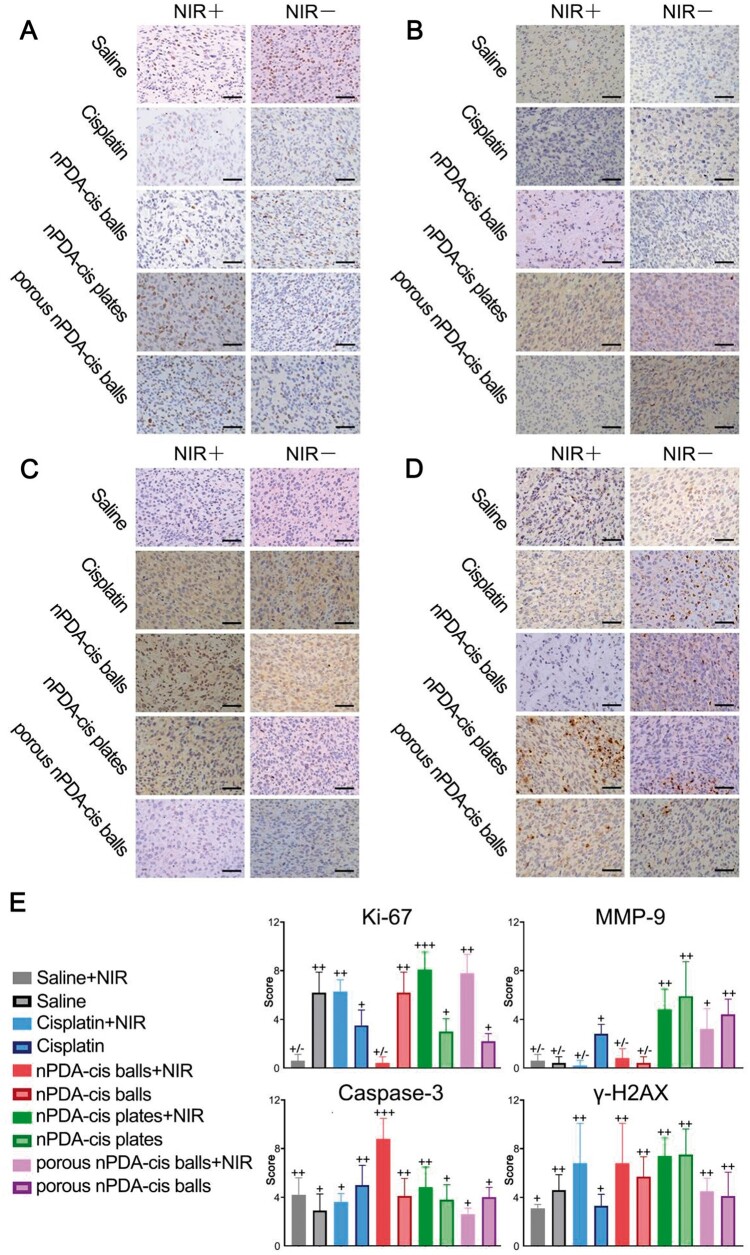
Gene expressions induced by different configurations of nPDA-cis, scale label = 50 μm. (**A**) Ki-67 immunohistochemical staining. (**B**) MMP-9 immunohistochemical staining. (**C**) Caspase-3 immunohistochemical staining. (**D**) γ-H2AX immunohistochemical staining. (**E**) The scores of immunohistochemical staining.

Further, we studied the *in vivo* changes in gene expressions induced by different configurations of nPDA-cis. nPDA-cis balls in combination with photothermal treatment had extremely low Ki-67 expression, indicating that nPDA-cis balls combined with NIR laser irradiation were able to inhibit tumor cell proliferation effectively (Figure 9A and E). Among the groups, nPDA-cis balls combined with NIR laser irradiation had the highest Ki-67 expression (Figure 9A and E), which was consistent with the largest tumor volume (Figure 8B).

As shown in Figure 9B and E, the expression of MMP-9 was very low in the saline group, indicating the weak invasive ability of SCC7 tumor cells (Figure 9B and E). And the nPDA-cis balls group also presented low MMP-9 expression, so it did not change this characteristic of weak invasive ability (Figure 9B and E). In contrast, the positive expression of MMP-9 was relatively strong in nPDA-cis plates and porous nPDA-cis balls, indicating that these two configurations of nPDA-cis may have the risk of promoting cancer cell invasion and metastasis (Figure 9B and E).

Caspase-3 expression (Figure 9C) in nPDA-cis balls with NIR irradiation group was significantly higher than that in the group without combination with NIR, indicating that the photothermal effect had a good synergistic effect with nPDA-cis balls, which promoted cancer cell apoptosis in mice (Figure 9C and E). The expression of Caspase-3 in the remaining groups was relatively close (Figure 9C and E).

As shown in Figure 9D, the expression of γ-H2AX was higher in animals exposed to nPDA-cis balls with NIR irradiation, indicating serious DNA damage in this group, which was consistent with the strong expression of Caspase-3 (Figure 9D and E). In nPDA-cis plates and porous nPDA-cis groups, the moderate positivity of γ-H2AX may be caused by the presence of tiny foci of necrosis within large tumors.

## Discussion

### Proliferation

In the proliferation assay, when comparing the experimental groups that combined photothermal treatment with those that did not, the group with combined photothermal treatment had a stronger ability to inhibit tumor cell proliferation. For the nPDA-cis balls with excellent therapeutic effect in live-dead staining experiments, the heat generated by the photothermal effect was the most important reason for their improved efficacy.

DNA is the critical target for cisplatin cytotoxicity. By binding to the N7 reactive center on purine residues, cisplatin can cause DNA damage in cancer cells, blocking cell division and resulting in apoptotic cell death. This process is related to the cell cycle, and only a few cells are affected in a short period of time. Besides, cisplatin itself has no photothermal effect [11]. So, for the cisplatin groups, it can be seen that the difference between the groups with or without NIR is smaller than that between the nPDA-cis groups.

The toxic effect of heat therapy on cells is divided into two phases; the first phase is a linear growth arrest, and the second phase is exponential cell death. The dose of thermal energy required for both phases is a function of the thermal treatment temperature and the corresponding thermal treatment duration. The first of these phases often occurs when the thermotherapy temperature is below 42°C to 43°C, and this phase of growth arrest is generally reversible. In contrast, the thermal dose required to enter the second phase is much higher and is closely related to the thermal dose required for cellular protein denaturation, with some studies reporting the need for 140 kcal/mol [31–33]. We believe that for nPDA-cis balls, the thermal energy dose produced by the photothermal effect has reached the threshold of the exponential cell death thermal energy dose. Therefore, in our experiments, we found that the photothermal effect of three different configurations of nPDA particles was able to effectively generate high temperatures to kill cells rapidly through cytotoxic effects. And the hyperthermia-induced acute cytotoxicity of nPDA-cis balls was particularly pronounced.

Hyperthermia can enhances the cytotoxicity of various antineoplastic agents, which is also called thermal chemosensitization. Some studies indicated that the platinum compounds are linearly enhanced in their cytotoxic effect when temperatures are raised from 37 to above 40.5°C. Heat is not able to cause severe DNA damage by itself, but instead hinders the repair of sublethal cell damage caused by cisplatin. This may be caused by a temperature-dependent inhibition of DNA-repair enzymes [5].

All in all, the nPDA-cis balls showed the best effect on inhibiting cell proliferation. The uneven temperature increase and more unstable treatment with plates and porous nPDA-cis balls were less effective and weaker than that with nPDA-cis balls in inhibiting proliferation. These results were consistent with the performance of the photothermal heating curve and repeated heating and also proved the synergistic effect of the photothermal effect on enhancing cytotoxicity from another perspective.

### Migration and invasion

For cisplatin and nPDA-cis balls, NIR laser irradiation was able to promote the inhibition of migration ability, where nPDA-cis balls inhibited migration stronger than cisplatin, which could be attributed to the synergistic effect of photothermal effect and chemotherapy. However, for the other groups of nPDA and nPDA-cis, the effect of photothermal effect on migration was not clear. Meanwhile, the effect of photothermal effect on the invasive ability of nPDA and nPDA-cis in our experiments was also unclear. The effect of nPDA-cis on the invasive ability of tumor cells was mainly related to the amount of cisplatin release. Notably, these results suggest that the effect of heat stress on the migration and invasion ability of tumor cells may be dual in nature.

It has been shown that heat therapy can inhibit tumor cell migration [34], and in the combination of heat therapy and chemotherapy, the heat effect also enhances the inhibition of tumor cell migration ability [35]. Different teams have reported that heat therapy inhibits the invasive ability of human tongue squamous carcinoma cells [36, 37]. Similar studies have been conducted for liver cancer and gastric cancer, and the related molecular mechanisms have been initially explored [34, 38]. In the field of head and neck tumors, the effect of heat therapy on the migration ability of human tongue squamous carcinoma may be related to TWIST2 protein, an important transcription factor for epithelial-mesenchymal transition, which has an important role in the malignant progression of tumors [39]. These studies suggest that thermotherapy may be effective in inhibiting the malignant progression of head and neck squamous carcinoma.

However, there are also many experiments showing that heat-shock proteins (HSPs) may also promote tumor cell migration and invasion given that heat stress leads to the activation of reparative pathways such as HSPs. For example, HSP90 promotes epithelial-mesenchymal transformation, invasion and migration [40, 41], and HSP90α secreted extracellularly also promotes cell motility [42, 43]. It has also been shown that HSP90α and HSP70 secreted by tumor cells promote the activation of matrix metalloproteinase 2 (MMP-2), thereby promoting invasion and migration [44]. In hepatocellular carcinoma, increased expression of HSP27 also promotes migration and invasion of cancer cells [45]. These results suggest that the role of heat therapy or heat stress and its synergy with chemotherapy in the migration and invasion of head and neck tumor cells and epithelial-mesenchymal transformation of tumor cells is complex. Our experiments also confirmed the existence of this duality, and the related mechanisms are yet to be investigated in depth.

### Apoptosis

Upon entry into cells, cisplatin can lead to apoptotic death via endogenous pathways centered on mitochondria, death receptor-mediated exogenous pathways and endoplasmic reticulum stress pathways, which ultimately cause a Caspase cascade reaction and execute apoptotic death via a Caspase-3-based terminal shear enzyme [46].

The specific mechanisms are related to three major apoptotic signaling pathways [46, 47]. In the endogenous pathway, cisplatin induces apoptosis by causing DNA damage, allowing phosphorylation and activation of p53, followed by cytochrome C release from mitochondria into the cytoplasm; meanwhile, cisplatin can also induce apoptosis in the endogenous pathway by inducing mitochondrial dysfunction and increasing reactive oxygen species production [48, 49]. In the exogenous pathway, cisplatin can lead to increased expression levels of the ligand fatty acid synthase (Fas) of the death receptor, tumor necrosis factor-α (TNF-α), and the death receptor itself, ultimately activating downstream proteases such as Caspase-3 that perform apoptosis [50, 51]. In the endoplasmic reticulum stress pathway, activation of calpain, which is localized on the cytoplasmic surface of the endoplasmic reticulum, is an early event in cisplatin-induced stress and is considered a non-nuclear target of cisplatin to induce apoptosis in the absence of DNA damage [52–54].

In contrast, the mode of cell death after heat stress is temperature and treatment time-dependent. Most data suggest that apoptosis due to heat treatment kills cells by activating the endogenous apoptotic pathway and that Caspase-2 upstream of Caspase-3 plays an important role in heat stress-induced cell death [55, 56]. In addition to the endogenous pathways, heat therapy also affects the exogenous apoptotic pathways by activating death receptors on the cell surface, and in some tumor cells, heat therapy induces apoptosis through Fas signaling and can activate both Caspase-3 and Caspase-8 [57, 58]. Therefore, the extent of apoptosis and apoptotic pathways differ between tumor cell types [59].

Stress signals that lead to cellular injury, while triggering the apoptotic cascade response, also induce stress responses, such as the expression and release of HSPs. In tumor cells, high expression of HSPs promotes autonomous cell proliferation, inhibits death pathways, plays an important role in tumor growth, and is closely associated with poor prognosis and chemotherapy resistance [60, 61]. Stress-induced HSPs, especially HSP27, 70 and 90, can play a key role in inhibiting apoptosis by interacting with important proteins and enzymes in the multiple apoptotic pathways described previously [45]. In contrast, Tang *et al.* [62] reported that tumor cells, including oral cancer cells, are resistant to heat therapy due to the following reason: in tumor cells, Caspase-3, the most critical apoptosis execution molecule, is not activated by heat stress. The interaction of the above two processes determines whether the final fate of the cell is the recovery from injury, further proliferation or death [63].

In our apoptosis experiments, NIR laser irradiation predominantly promoted apoptosis in the cisplatin group without photothermal effects and in the blank control group, indicating the dominance of the apoptotic cascade response in these groups and the absence of defects in the apoptosis-related pathways caused by cisplatin in the tumor cell lines we used. In contrast, in the three constitutive nPDA-cis experimental groups, the stress response process may dominate and thus apoptosis is inhibited within a short period of time, which is consistent with the secretory characteristics of HSPs after heat stress in *in vitro* studies.

The above results were obtained with a shorter incubation time, so the results were less affected by the effect of cisplatin on apoptosis. Considering the longer duration of drug action in practical application and the more complex *in vivo* microenvironment, the effect of heat stress, cisplatin induction of apoptosis at different time points, and the mechanism of action need to be further investigated and explored in depth.

### Drug resistance *in vivo*

The previously mentioned duality of the effect of thermotherapy on tumors becomes more complicated in the *in vivo* setting. In the *in vivo* experiments, we found that the treatment effect of nPDA-cis balls was superior to that of the cisplatin group, while the effect of nPDA-cis balls combined with photothermal treatment was significantly better than that of the cisplatin combined with photothermal treatment group, which was different from the *in vitro* experiments. We suggest that the photothermal effect of nPDA-cis balls produced a synergistic effect with the chemotherapeutic action of cisplatin *in vivo*, likely caused by the hemodynamic and immune system antitumor immune enhancement, which eventually led to the activation of apoptotic pathways as the dominant determinant of the fate of the cells.

Unlike tumor cells cultured *in vitro*, tumor tissue in living organisms is influenced by vascular structure, hemodynamics, microenvironment and the immune system. In animal experiments, we chose C3H mice, which have a healthy immune system, instead of the commonly used immunodeficient nude mice, to better reflect real-world scenarios. The immune system plays a very complex role in the development and treatment of HNSCC, and is also affected by hyperthermia and chemotherapy. Heat therapy can alter the local microenvironment and affect drug distribution by dilating local blood vessels, and furthermore, heat therapy has a significant effect on the immune system, which can promote the killing of tumor cells. For example, heat therapy can activate NK cells and enhance the immunogenicity of tumor cells [6, 64, 65]. In both tumor-bearing mice and oral cancer patients, heat therapy results in elevated lymphocyte transformation index (LTI), CD4+ cell counts, and significantly increased levels of interleukin-2 (IL-2) and tumor necrosis factor-α (TNF-α) activity [66]. In oral squamous carcinoma, photothermal treatment induces apoptosis in tumor cells while promoting their expression and release of damage-related molecular patterns that enhance the immunogenicity of tumor cells as an endogenous danger signal and further mediates antitumor immune responses [67].

The previously mentioned HSPs are a family of highly conserved stress-inducible proteins that attenuate the effects of stress on cells during homeostasis and have potent anti-apoptotic properties [68, 69]. HSPs are expressed at higher levels in many tumors than in normal tissues, and the immune system recognizes HSPs as ‘danger signals’ to enhance immune responses [70]. Heat therapy leads to an enhanced immune response to the tumor in the body by molecular mechanisms that include the production of HSPs, activation of antigen-presenting cells, and altered lymphocyte transport [6]. Therefore, despite the considerable complexity of the clinical applications and underlying mechanisms of heat therapy and heat chemotherapy, the synergistic multimodal treatment of heat therapy, heat chemotherapy and immunotherapy remains an important direction for future drug development.

## Conclusion

In this study, we successfully designed and synthesized three different configurations of nPDA-cis drug carrier systems, including balls, plates and porous balls, and compared their physical and chemical properties, and their effects on cancer cells *in vitro* and *in vivo*. Among them, the nPDA-cis balls drug carrier system combined with photothermal therapy was equivalent to or better than cisplatin alone at each time point *in vivo* and *in vitro*, and had no obvious toxicity *in vivo*, which may have good clinical application prospects. The different therapeutic effects of nPDA-cis of the three different configurations indicate that it is necessary to study the effect of chemical structure on function in future drug development and drug delivery system development. They also suggest that the microstructure design of polymeric materials may be an important research direction for the development of new therapeutic methods. In addition, the immune system *in vivo* and the physical and chemical properties of tissues may also affect the therapeutic effect, which should be paid attention to in future studies. The synergy between hyperthermia and immunotherapy provides a direction for drug development in the future. Our experiments preliminarily confirmed that hyperthermia in combination with chemotherapy was superior to chemotherapy alone. We propose that immunotherapy can work in concert with hyperthermia and chemotherapy in the future to provide more minimally invasive and effective options for the treatment of HNSCC.

## Supplementary Material

rbae073_Supplementary_Data
