# Supplementary material for: The therapeutic efficacy of different configuration nano-polydopamine drug carrier systems with photothermal synergy against head and neck squamous cell carcinoma
Source: Regen Biomater. 2024 Jun 20;11:rbae073. doi: 10.1093/rb/rbae073 (PMC11256922; doi:10.1093/rb/rbae073)
Supplement: rbae073_Supplementary_Data [file rbae073_supplementary_data.zip › Gyh-Supporting Information for RB-20240603.docx]

**Supporting Information**

**Synthesis of nPDA and nPDA-cis**

The chemical reagents used in the synthetic process are listed in Table 1 below.

**Preparation of nPDA balls:** 250 mg of DA-HCl was weighed and added to a 250 mL round-bottom flask, followed by 100 mL of ultrapure water. The mixture was shaken until the solid completely dissolved. The flask was then fixed on a magnetic stirrer and stirring began. 40 mL of anhydrous ethanol was added, followed by quickly adding 0.5 mL of ammonia water. The reaction was maintained at a constant temperature of 37℃, with continuous stirring for 8-12 hours. After the reaction, the solution was collected in a 100 mL centrifuge tube and centrifuged at 5-10℃, 10000 rpm for 10 minutes using a refrigerated centrifuge.

**Preparation of nPDA plates:** 200 mg of DA-HCl was weighed and added to a 250 mL round-bottom flask, followed by 90 mL of ultrapure water. The mixture was shaken until the solid completely dissolved. The flask was then fixed on a magnetic stirrer with a magnetic stirring bar and stirring began. The reaction was maintained at a constant temperature of 70℃, with continuous stirring for 30 hours. After the reaction, the solution was collected in a 100 mL centrifuge tube and centrifuged at 5-10℃, 10000 rpm for 10 minutes using a refrigerated centrifuge.

**Preparation of porous nPDA balls:** 25 mg P123, 75 mg F127, 150 mg DA-HCl, and 0.4 mL TMB were weighed and added to a previously prepared mixture of 5 mL ultrapure water and 5 mL ethanol. The mixture was mixed evenly using ultrasound for 1 minute. The reaction vessel was fixed to a magnetic stirrer and stirring began, followed by adding 0.2 mL ammonia water. The reaction was maintained at a constant temperature of 25℃, with continuous stirring for 2 hours. After the reaction, the solution was collected in a 100 mL centrifuge tube and centrifuged at a temperature of 5-10℃, 10000 rpm for 10 minutes using a refrigerated centrifuge.

In all three different synthetic reactions, the solution was observed to change from colorless and transparent to the dark color caused by PDA. During the synthesis of nPDA balls, after adding ammonia water, the solution quickly turned dark brown; during the synthesis of nPDA plates, the solution gradually turned earthy yellow; during the synthesis of porous nPDA balls, the solution gradually turned turbid brown. After centrifugation of the aforementioned reaction solutions, the supernatant was discarded, and the precipitate was resuspended in ultrapure water, then centrifuged again under the same conditions, repeating 4-5 times until the supernatant was completely clear. Collect the precipitate in a 15 mL centrifuge tube, adjust to a volume of 10 mL, and store in a 4℃ refrigerator for future use.

**Preparation of nPDA-cis particles:** The solutions of the three different configurations of nPDA particles mentioned above were taken, and cisplatin drug was added to each of the three different configurations of nPDA particle solutions according to the different mass ratios of cisplatin to nPDA particles shown in Table 2. The volume was made up to 10 mL with ultrapure water to achieve the final concentrations indicated in Table 2. The mixture was stirred with a magnetic stirrer at 37℃ in a light-protected environment for 24 hours to complete the drug loading synthesis of the nPDA particles. The final reaction liquid was collected as the successfully constructed nPDA-cis.

The different mass ratios of cisplatin to nPDA particles is shown in Table 2.

Table 1. Main Reagents Used for the Preparation of nPDA and nPDA-cis

| Reagent Name | Purity Grade | Company | Country |
| --- | --- | --- | --- |
| Dopamine Hydrochloride (DA-HCl) | Analytical Grade | Sinopharm | China |
| Anhydrous Ethanol | Analytical Grade | Sinopharm | China |
| Ammonia Water (23%~28% mass fraction) | Analytical Grade | Sinopharm | China |
| P123 (EO20PO70EO20)* | Analytical Grade | Sigma-Aldrich | USA |
| F127 (EO106PO70EO106)* | Analytical Grade | Sigma-Aldrich | USA |
| Trimethylbenzene (TMB) | Analytical Grade | Sigma-Aldrich | USA |
| Cisplatin | Chromatographic Grade | Sigma-Aldrich | USA |

*: The reagents P123 and F127 in the table are amphiphilic triblock copolymers formed by ethoxy and propoxy groups, but the block compositions are different; EO denotes ethoxy group, PO denotes propoxy group.

Table 2. Experimental Grouping for nPDA Loaded with Cisplatin

| Group Number | Cisplatin  (Mass Ratio) | Final Concentration of Cisplatin (mg/mL) | Final Concentration of nPDA (mg/mL) | nPDA Configuration |
| --- | --- | --- | --- | --- |
| 1 | 120% | 2.4 | 2 | Balls |
| 2 | 80% | 1.6 | 2 | Balls |
| 3 | 40% | 0.8 | 2 | Balls |
| 4 | 20% | 0.4 | 2 | Balls |
| 5 | 120% | 2.4 | 2 | Plates |
| 6 | 80% | 1.6 | 2 | Plates |
| 7 | 40% | 0.8 | 2 | Plates |
| 8 | 20% | 0.4 | 2 | Plates |
| 9 | 120% | 2.4 | 2 | Porous balls |
| 10 | 80% | 1.6 | 2 | Porous balls |
| 11 | 40% | 0.8 | 2 | Porous balls |
| 12 | 20% | 0.4 | 2 | Porous balls |

**Drug Safety Evaluation**

The body weight curve of mice shows in the main document Fig. 8F can reflect the safety of drugs. For the group with the best treatment effect, which is nPDA-cis balls with NIR irradiation group therapy, there is no statistically significant difference between the body weight of this experimental group and that of the saline control group, which can be considered as a safe way of treatment.

In order to preliminarily understand the safety of three configurations of nPDA-cis with or without NIR irradiation, we collected organ tissue samples from C3H mice on day 10 for a comparative study of morphological changes in the heart, liver, spleen, lung, and kidney. Hematoxylin and eosin staining of tissue slices was performed and imaged using a Leica microscope, as shown in Figure 1 and 2. As a result, HE staining of organs in mice from each group showed no significant morphological abnormalities. It can be preliminarily determined that all constructions of nPDA-cis are relatively safe drug carrier systems.
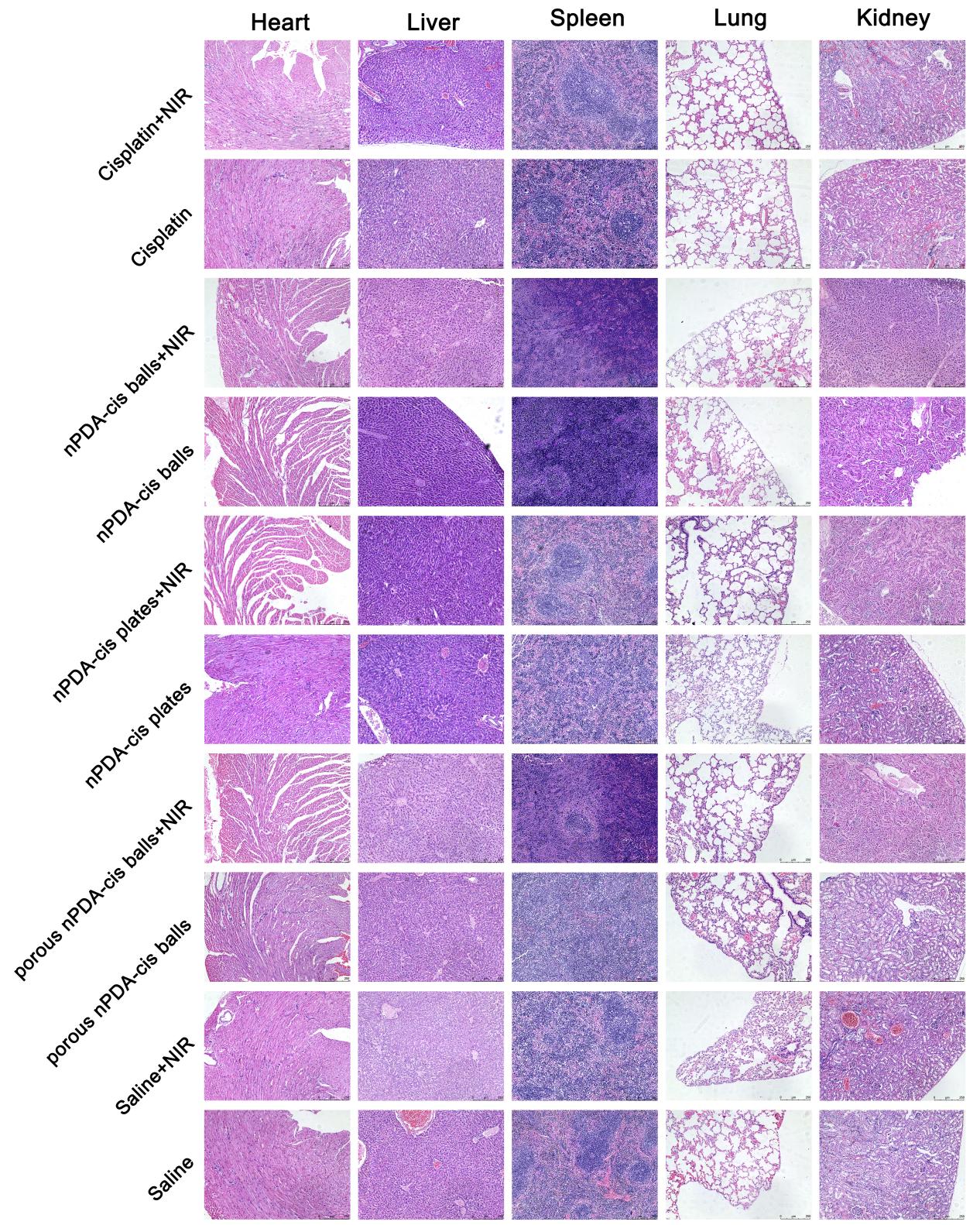


**Figure 1.** Histology of of tissue organs in tumor-bearing mice.


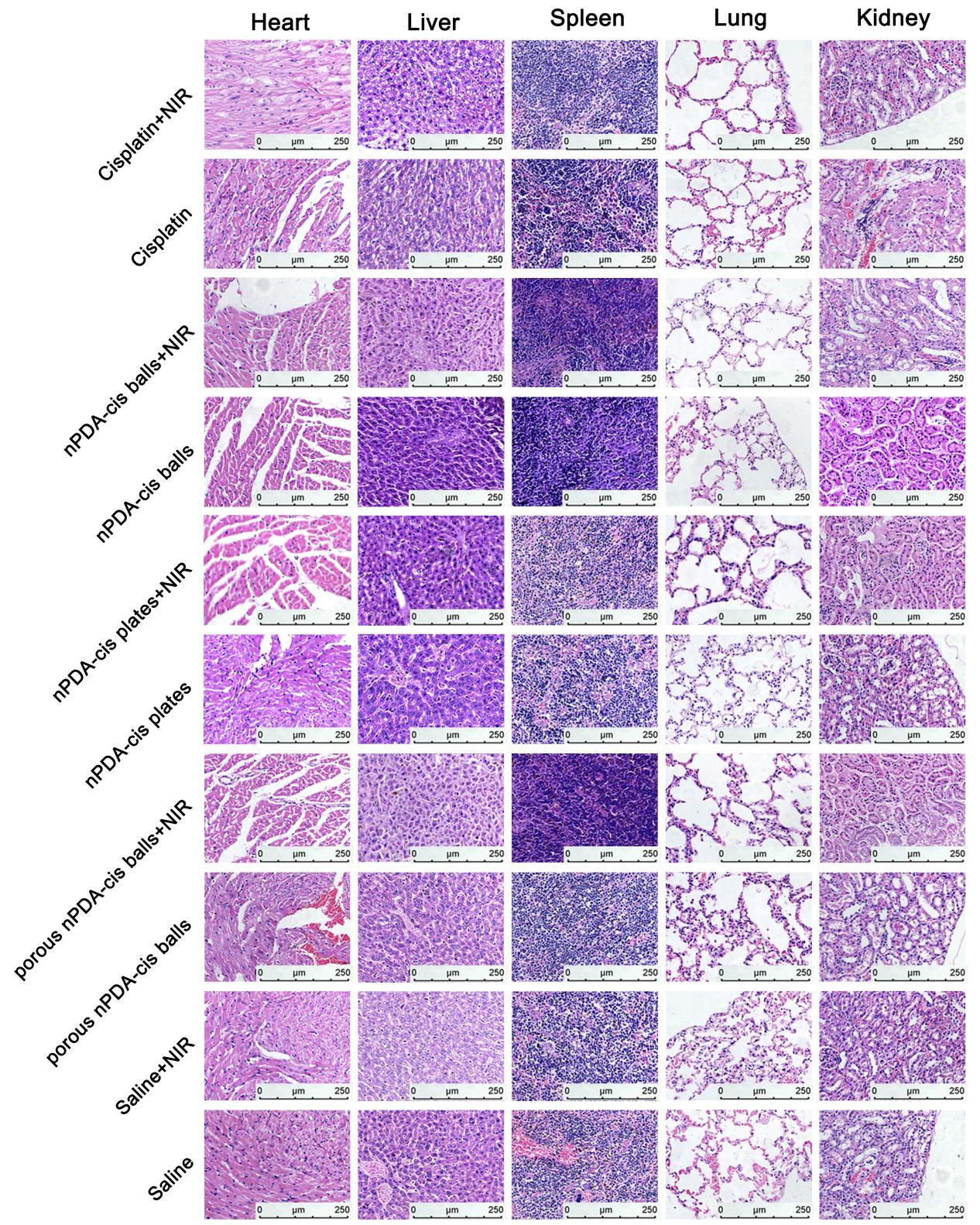


**Figure 2.** Histology of of tissue organs in tumor-bearing mice.
