# Supplementary material for: The therapeutic efficacy of different configuration nano-polydopamine drug carrier systems with photothermal synergy against head and neck squamous cell carcinoma
Source: Regen Biomater. 2024 Jun 20;11:rbae073. doi: 10.1093/rb/rbae073 (PMC11256922; doi:10.1093/rb/rbae073)
Supplement: rbae073_Supplementary_Data [file rbae073_supplementary_data.zip › Gyh-Responses to review comments for RB-20240603.docx]

**Responses to review comments**

Manuscript ID: RB-2024-130

Manuscript Title: The Therapeutic Efficacy of Different Configuration Nano-polydopamine Drug Carrier Systems with Photothermal Synergy against Head and Neck Squamous Cell Carcinoma

Article Type: Original research paper

**Dear Editors and Reviewers:**

Thank you very much for your letter and for the reviewers' comments on our manuscript. These comments are all valuable and very helpful for improving the quality of this paper. We have studied them carefully and made revisions based on the comments of the reviewers. The primary corrections in the manuscript and the responses to the comments are shown point-to-point as follows. In the revised paper, all these alterations, as well as others which are made to improve the paper but will not affect its content and framework, are marked in red. We are looking forward to your positive response. If any further clarification is needed, please contact me. Thank you very much.

Yours sincerely,

**Yuhao Guo**

**Bo Li**

**Kexin Yao**

**Longjiang Li**

(on behalf of all authors)

State Key Laboratory of Oral Diseases & National Clinical Research Center for Oral Diseases, Department of Head and Neck Oncology, West China Hospital of Stomatology, Sichuan University, Chengdu 610041, PR China.

E-mail: muzili63@163.com; Phone: 86-28-85501440; Fax: 86-28-85582167

**Reviewer #1:**

Comments to the Author

The manuscript, "The Therapeutic Efficacy of Nano-polydopamine Drug Carrier Systems in Various Configurations with Photothermal Synergy against Head and Neck Squamous Cell Carcinoma," investigates nPDA's utility in different shapes as cisplatin carriers. It aims to improve HNSCC treatment by combining chemotherapy with photothermal therapy. The authors synthesized nPDA in three shapes: spherical, plate-like, and porous spherical. They then loaded these structures with cisplatin, creating nPDA-cis systems, to evaluate their physicochemical properties, photothermal effects, and drug delivery capabilities, alongside their effectiveness in combating cancer both in vitro and in vivo. Despite the manuscript's focus on employing polydopamine nanoparticles for drug delivery and photothermal therapy, this area has been extensively researched. The lack of substantial innovations or insights makes it challenging to establish the study's significance and relevance in a competitive domain. Some major points should be noticed and addressed in the future submission:

**Major comments:**

**(1)The manuscript provides inadequate details on the synthesis and specific characteristics of the nanoparticle shapes (spherical, plate-like, and porous spherical).**

We appreciate your valuable advice. We regret that due to the for lack of space, our original manuscript described the synthesis of three types of nPDA and three types of nPDA-cis rather briefly. The chemical reagents used in the synthetic process are listed in Table 1 below.

**Table 1.** Main Reagents Used for the Preparation of nPDA and nPDA-cis

| **Reagent Name** | **Purity Grade** | **Company** | **Country** |
| --- | --- | --- | --- |
| Dopamine Hydrochloride (DA-HCl) | Analytical Grade | Sinopharm | China |
| Anhydrous Ethanol | Analytical Grade | Sinopharm | China |
| Ammonia Water (23%~28% mass fraction) | Analytical Grade | Sinopharm | China |
| P123 (EO20PO70EO20)* | Analytical Grade | Sigma-Aldrich | USA |
| F127 (EO106PO70EO106)* | Analytical Grade | Sigma-Aldrich | USA |
| Trimethylbenzene (TMB) | Analytical Grade | Sigma-Aldrich | USA |
| Cisplatin | Chromatographic Grade | Sigma-Aldrich | USA |

*: The reagents P123 and F127 in the table are amphiphilic triblock copolymers formed by ethoxy and propoxy groups, but the block compositions are different; EO denotes ethoxy group, PO denotes propoxy group.

Specifically, these six kinds of materials were obtained as follows:

For the preparation of nPDA balls: 250 mg of DA-HCl was weighed and added to a 250 mL round-bottom flask, followed by 100 mL of ultrapure water. The mixture was shaken until the solid completely dissolved. The flask was then fixed on a magnetic stirrer and stirring began. 40 mL of anhydrous ethanol was added, followed by quickly adding 0.5 mL of ammonia water. The reaction was maintained at a constant temperature of 37℃, with continuous stirring for 8-12 hours. After the reaction, the solution was collected in a 100 mL centrifuge tube and centrifuged at 5-10℃, 13000 rpm for 10 minutes using a refrigerated centrifuge.

For the preparation of nPDA plates: 200 mg of DA-HCl was weighed and added to a 250 mL round-bottom flask, followed by 90 mL of ultrapure water. The mixture was shaken until the solid completely dissolved. The flask was then fixed on a magnetic stirrer with a magnetic stirring bar and stirring began. The reaction was maintained at a constant temperature of 70℃, with continuous stirring for 30 hours. After the reaction, the solution was collected in a 100 mL centrifuge tube and centrifuged at 5-10℃, 10000 rpm for 10 minutes using a refrigerated centrifuge.

For the preparation of porous nPDA balls: 25 mg P123, 75 mg F127, 150 mg DA-HCl, and 0.4 mL TMB were weighed and added to a previously prepared mixture of 5 mL ultrapure water and 5 mL ethanol. The mixture was mixed evenly using ultrasound for 1 minute. The reaction vessel was fixed to a magnetic stirrer and stirring began, followed by adding 0.2 mL ammonia water. The reaction was maintained at a constant temperature of 25℃, with continuous stirring for 2 hours. After the reaction, the solution was collected in a 100 mL centrifuge tube and centrifuged at a temperature of 5-10℃, 10000 rpm for 10 minutes using a refrigerated centrifuge.

In all three different synthetic reactions, the solution was observed to change from colorless and transparent to the dark color caused by PDA. During the synthesis of nPDA balls, after adding ammonia water, the solution quickly turned dark brown; during the synthesis of nPDA plates, the solution gradually turned earthy yellow; during the synthesis of porous nPDA balls, the solution gradually turned turbid brown. After centrifugation of the aforementioned reaction solutions, the supernatant was discarded, and the precipitate was resuspended in ultrapure water, then centrifuged again under the same conditions, repeating 4-5 times until the supernatant was completely clear. Collect the precipitate in a 15 mL centrifuge tube, adjust to a volume of 10 mL, and store in a 4℃ refrigerator for future use.

Preparation of nPDA-cis particles: The solutions of the three different configurations of nPDA particles mentioned above were taken, and cisplatin drug was added to each of the three different configurations of nPDA particle solutions according to the different mass ratios of cisplatin to nPDA particles shown in Table 2. The volume was made up to 10 mL with ultrapure water to achieve the final concentrations indicated in Table 2. The mixture was stirred with a magnetic stirrer at 37℃ in a light-protected environment for 24 hours to complete the drug loading synthesis of the nPDA particles. The final reaction liquid was collected as the successfully constructed nPDA-cis.

The different mass ratios of cisplatin to nPDA particles is shown in Table 2.

**Table 2.** Experimental Grouping for nPDA Loaded with Cisplatin

| Group Number | Cisplatin  (Mass Ratio) | Final Concentration of Cisplatin (mg/mL) | Final Concentration of nPDA (mg/mL) | nPDA Configuration |
| --- | --- | --- | --- | --- |
| 1 | 120% | 2.4 | 2 | Balls |
| 2 | 80% | 1.6 | 2 | Balls |
| 3 | 40% | 0.8 | 2 | Balls |
| 4 | 20% | 0.4 | 2 | Balls |
| 5 | 120% | 2.4 | 2 | Plates |
| 6 | 80% | 1.6 | 2 | Plates |
| 7 | 40% | 0.8 | 2 | Plates |
| 8 | 20% | 0.4 | 2 | Plates |
| 9 | 120% | 2.4 | 2 | Porous balls |
| 10 | 80% | 1.6 | 2 | Porous balls |
| 11 | 40% | 0.8 | 2 | Porous balls |
| 12 | 20% | 0.4 | 2 | Porous balls |

The details of the preparation of nPDA and nPDA-cis mentioned above are described in the ***Supporting Information*** of this revised manuscript, for the understanding of reviewers and readers. And we have made the following modifications to the original text in Lines 148-149 and 159-168:

"The above reaction suspensions were collected and centrifuged at 5-10 and 13,000 r/min for 10 min."

**"Preparation of nPDA-cis particles:** According to the different mass ratios of cisplatin with nPDA particlesthe in different experiments, the cisplatin drug was added to the three different configurations of nPDA particles solutions respectively, and the volume of ultrapure water was replenished to 10 mL, so that the final concentration reached the requirements. The reaction was stirred at 37℃ at constant temperature in the dark for 24h to obtain the successful nPDA-cis particles."

"The chemical reagents used in the synthetic process are listed in *Supporting Information Table 1*. The different mass ratios of cisplatin to nPDA particles is shown in *Supporting Information Table 2*. More details of the preparation of nPDA and nPDA-cis mentioned above are described in the ***Supporting Information*** of this paper."

**(2) An in-depth examination of how the design of nanoparticles influences their therapeutic effectiveness is required.**

Thank you for your valuable comment. Yes, as the reviewer pointed out, the relationship between the design of nanoparticles and their therapeutic effects is very important, and this is a key aspect emphasized in this paper. Existing studies mostly focus on the particle size and surface properties of nanoparticles (such as surface charge and hydrophobicity, surface modifications of nanoparticles, etc.) [1-3]. However, comparative studies on the physicochemical properties of different configurations of nanomaterials synthesized from the same monomer, and the biological effects and other properties under the same experimental conditions are still relatively rare [4].

This paper aims to enhance the therapeutic efficacy of cisplatin for HNSCC through the synergistic effect of photothermal therapy. Based on the hypothesis that different spatial configurations of nPDA-cis might possess distinct physical, chemical, and biological properties, which could lead to varying therapeutic effects on HNSCC, we conducted experiments using the same dopamine monomer to design and synthesize three different spatial configurations of nPDA: nPDA balls, nPDA plates, and porous nPDA balls. These were successfully loaded with cisplatin to form nPDA-cis (nPDA-cis balls, nPDA-cis plates, and porous nPDA-cis balls). Subsequently, using nPDA and nPDA-cis, systematic analyses and tests were carried out on their morphology, chemical structure, element spatial distribution, photothermal properties, drug loading and release, biological interactions, therapeutic effectiveness, and in vivo performance.

This paper confirms that the three different configurations of nPDA (nPDA balls, nPDA plates, and porous nPDA balls) exhibit unique physical properties due to their shape and structure, which in turn influence their biological interactions and photothermal effects significantly. Our description of the unique results obtained in this study including:

Photothermal effect: Nanoparticles of different configurations exhibit exhibit distinct characteristics during the heating process under NIR irradiation. nPDA-cis balls and porous nPDA-cis balls demonstrate better uniformity and stability in temperature rise during NIR laser irradiation compared to nPDA-cis plates, which show less uniform and unstable temperature profiles. This implies that nPDA-cis balls and porous nPDA-cis balls could provide more effective and predictable photothermal therapy.

Drug Loading and Release: Different configurations of nanoparticles exhibit varying efficiencies in drug loading and release profiles. The nPDA balls were noted for higher encapsulation efficiency of cisplatin, especially as the mass ratio of cisplatin to nPDA increased, reaching stability at higher concentrations. On the other hand, nPDA plates and porous nPDA balls showed less ideal encapsulation and release behaviors, which could affect the overall therapeutic outcomes.Surface Characteristics and Biological Interactions: The interaction of nanoparticles with biological systems, including their ability to penetrate cancer cells, also relates to their design. For instance, the surface charge of nanoparticles changes with varying configurations and cisplatin loadings, influencing cellular uptake and distribution within the body.

Therapeutic Effectiveness: The efficacy of these nanoparticles in killing cancer cells and reducing tumor growth varied with their design. nPDA balls, particularly when combined with cisplatin (nPDA-cis balls), showed a synergistic effect with photothermal therapy, resulting in enhanced tumor cell killing both in vitro and in vivo. Conversely, nPDA plates and porous nPDA balls were less effective in certain scenarios, suggesting that their designs were less optimal for effective cancer therapy.

In Vivo Performance: In vivo experiments confirmed that the design of nPDA significantly affects therapeutic outcomes. nPDA-cis balls exhibited the best tumor inhibition, both with and without NIR laser irradiation, highlighting the importance of nanoparticle design in enhancing the efficacy of combined photothermal and chemotherapy treatments.

Overall, our study does demonstrate the critical role of nanoparticle design in determining their physical properties, interaction with biological systems, drug efficiency, and ultimately their therapeutic effectiveness, especially in the context of nano-polydopamine (nPDA) used as a drug carrier for HNSCC treatment.

Additionally, we have strengthened the expression of our unique conclusions in the Results section, Lines 441-445, as follows:

"In conclusion, nPDA-cis balls and porous nPDA-cis balls demonstrate better uniformity and stability in temperature rise during NIR laser irradiation compared to nPDA-cis plates, which show less uniform and unstable temperature profiles. This implies that nPDA-cis balls and porous nPDA-cis balls could provide more effective and predictable photothermal therapy."

**(3) The manuscript omits a comparative analysis that would highlight the unique advantages or innovations of nPDA configurations against current alternatives.**

Thank you for reminding us to enhance the comparative analysis between nPDA configuration and current alternatives in our manuscript, which would significantly improve the understanding of the unique advantages and innovations of nPDA configurations

Cisplatin is a classic chemotherapy drug and remains a commonly used chemotherapy drugs for HNSCC. For example, cisplatin 100 mg/m^2^ given once every 3 weeks is the standard of care in locally advanced head and neck squamous cell cancer, but there are significant treatment-related toxicities observed[5, 6]. At the same time, there were relatively few studies chose platinum compounds to design drug carrier systems. And past studies have used PDA as part of the surface modification of nanoparticles, each carrier system involving a variety of raw material monomers, and the synthesis and preparation process is complex [7-10]. In our experiment, considering the clinical safety issues, we aimed to use the minimum number of monomer types possible. Therefore, we chose PDA for its high biocompatibility. The unique properties of nPDA such as enhanced photothermal effects and customized drug carrier capabilities theoretically suggest significant advancements over traditional nanoparticles. These features are crucial for developing more targeted and less toxic therapeutic options, addressing some of the limitations of current nanoparticles like non-specificity and systemic toxicity. Since only one monomer is used, our synthesis process is relatively simple, which facilitates control of variables and subsequent scale-up of production.

Furthermore, in animal experiments, we chose C3H mice, which have a robust immune system, instead of the commonly used immunodeficient nude mice, to better reflect real-world scenarios. The immune system plays a very complex role in the development and treatment of HNSCC. The emergence of new cancer immunotherapy, such as immune checkpoint inhibitors, has already highlighted this point. However, previous studies using nude mice may have overlooked the important role of this system [10]. Due to the complexity of the immune system's role, controlling variables is necessary. Hence, in the initial design of the drug carrier system, we chose to use only PDA as a monomer to avoid the immune system's response caused by introducing more different substances, and focused on how changes pf configurations affect therapeutic efficacy.

In our study, we focused on detailing the physicochemical properties of three different nPDA configurations (nPDA balls, plates, and porous balls) and the potential of photothermal synergy to enhance the therapeutic effects of cisplatin. Although we did not provide a direct comparative experiment with the existing nanoparticle systems, we highlighted their high photothermal conversion efficiency and effective drug encapsulation and release capabilities. Indirectly positioning nPDA as a promising candidate whose design flexibility is not always feasible in other nanoparticle systems.

We believe that a detailed comparative study with established nanoparticle systems could be a valuable extension of this work. As part of our ongoing research efforts, we are exploring collaborations with other researchers to initiate side-by-side comparisons in clinical settings to quantitatively evaluate the advantages of nPDA configurations over existing alternatives. These future endeavors will aim to robustly demonstrate the practical benefits of nPDA and solidify its place in advanced nanoparticle therapies.

We appreciate your comment, as it underscores a critical avenue for expanding the impact of our research and contributing more definitively to the field of nanomedicine. And in the revised manuscript's discussion section, Lines 863-867, we added:

"In animal experiments, we chose C3H mice, which have a healthy immune system, instead of the commonly used immunodeficient nude mice, to better reflect real-world scenarios. The immune system plays a very complex role in the development and treatment of HNSCC, and is also affected by hyperthermia and chemotherapy."

**(4) Although the manuscript addresses in vivo efficacy, it fails to evaluate the safety profiles of the nPDA configurations.**

Thank you for your experience and concern regarding the safety of drug carrier systems. Yes, any new drug or drug carrier system must undergo rigorous safety assessments before being used clinically. This paper does not present a safety discussion for the following reasons:

First, when designing the experiments, it was hypothesized that different spatial configurations of nPDA-cis might have different physical, chemical, and biological properties, thereby having different therapeutic effects on HNSCC. This study is still in the laboratory validation stage of comparing therapeutic effects and testing hypotheses, and it is still some distance from clinical application.

Second, in the selection of base materials used for synthesizing nPDA-cis, we considered the safety issues of the materials; cisplatin is an antitumor drug already used clinically. Dopamine, also known as 3,4-dihydroxyphenethylamine, is an endogenous nitrogen-containing compound. Polydopamine, formed from the self-assembly of dopamine monomers, is a polymer widely used in the biomedical and food industries [11-15].

Third, during the animal experiment process, we conducted preliminary safety-related experiments. The body weight curve of mice can reflect the safety of drugs (Fig. 8F). For the group with the best treatment effect, which is nPDA-cis balls with NIR irradiation group therapy, there is no statistically significant difference between the body weight of this experimental group and that of the saline control group, which can be considered as a safe way of treatment.

More results and discussions about safety were not included in the main text due to space limitations, and we will present preliminary results related to safety in the ***Supporting Information***.

As with the reviewer's concerns about the safety of nPDA, before this drug carrier system enters clinical trials, we will strictly follow various guidelines issued by the Center for Drug Evaluation of the National Medical Products Administration, such as the "Guidance for Evaluation of the Safety and Efficacy of Medical Devices Using Nano Materials", "Technical Guidelines for the Preparation of Safety Summary Documents for Antitumor Innovative Drug Marketing Applications" and so on to conduct research and evaluation studies related to safety.

**(5) The study lacks an exploration of the underlying mechanisms. Investigating how photothermal effects enhance cisplatin's cellular and molecular activity would add depth to the research.**

Thank you for your valuable comment and suggestions. We have made the following additions in the discussion section of the revised manuscript, Lines723-760:

"In the proliferation assay, when comparing the experimental groups which combined photothermal treatment with those which did not, the group with combined photothermal treatment had a stronger ability to inhibit tumor cell proliferation. For the nPDA-cis balls with excellent therapeutic effect in live-dead staining experiment, the heat generated by the photothermal effect was the most important reason of their improved efficacy.

DNA is the critical target for cisplatin cytotoxicity. By binding to the N7 reactive center on purine residues, cisplatin can cause DNA damage in cancer cells, blocking cell division and resulting in apoptotic cell death. This process is related to the cell cycle, and only a few cells are affected in a short period of time. Besides, cisplatin itself has no photothermal effect [11]. So for the cisplatin groups, it can be seen that the difference between the groups with or without NIR is smaller than that between the nPDA-cis groups.

The toxic effect of heat therapy on cells is divided into two phases; the first phase is a linear growth arrest, and the second phase is exponential cell death. The dose of thermal energy required for both phases is a function of the thermal treatment temperature and the corresponding thermal treatment duration. The first of these phases often occurs when the thermotherapy temperature is below 42 to 43℃, and this phase of growth arrest is generally reversible. In contrast, the thermal dose required to enter the second phase is much higher and is closely related to the thermal dose required for cellular protein denaturation, with some studies reporting the need for 140 kcal/mol [31-33]. We believe that for nPDA-cis balls, the thermal energy dose produced by the photothermal effect has reached the threshold of the exponential cell death thermal energy dose. Therefore, in our experiments, we found that the photothermal effect of three different configurations of nPDA particles was able to effectively generate high temperature to kill cells rapidly through cytotoxic effects. And the hyperthermia-induced acute cytotoxicity of nPDA-cis balls was particularly pronounced.

Hyperthermia can enhances the cytotoxicity of various antineoplastic agents, which is also called thermal chemosensitization. Some studies indicated that the platinum compounds are linearly enhanced in their cytotoxic effect when temperatures are raised from 37 to above 40.5℃. Heat is not able to cause severe DNA-damage by itself, but instead hinders the repair of sublethal cell damage caused by cisplatin. This may be caused by a temperature-dependant inhibition of DNA-repair enzymes [5].

All in all, the nPDA-cis balls showed the best effect on inhibiting cell proliferation. The uneven temperature increase and more unstable treatment with plates and porous nPDA-cis balls was less effective and weaker than that with nPDA-cis balls in inhibiting proliferation. These results was consistent with the performance of the photothermal heating curve and repeated heating, and also proved the synergistic effect of photothermal effect on enhancing cytotoxicity from another perspective."

As discussed above, the mechanism by which the photothermal effect affects the therapeutic effectiveness of cisplatin and nPDA-cis in treating tumors is very complex and far from being fully clear in this paper. Fortunately, we are conducting experimental research on the competition and utilization of photothermal energy among nPDA, cisplatin, and cancer cells from the dimensions of material isothermal titration microcalorimetry, point free energy, cell thermodynamics, and biochemical reaction kinetics, in order to explain how the photothermal effect enhances the efficacy of cisplatin. This will be another paper of ours, and the reviewer's suggestions will be of positive help to our ongoing work.

**Reviewer: 2**

Comments to the Author

In this study, the authors designed and synthesized three different configurations of nPDA-cis drug carrier systems with good photothermal effects, and compared their effects on cancer cells in vitro and in vivo. Not only did it present a drug with potential good clinical application prospects, but also mentioned the inconsistency between in vivo and in vitro phenomena. The mention of a certain duality suggests a very interesting research direction. In general, the article is quite innovative and could be accepted for publication after the following points are addressed:

**Major comments:**

**（1）Suggestion for native English speakers to polish, for example: In the abstract part: "Carriers with photothermal effect are the drug carrier systems that can promote the killing of cancer cells and help overcome drug resistance through heat stress" has poor word order. I recommend that the author revise it to "The drug carrier systems with photothermal effect can promote the killing of cancer cells and help overcome drug resistance through heat stress."**

**In the introduction part: "Therefore, utilizing the synergistic effect of chemotherapy and hyperthermia has also become an important direction for the development of new drugs." I recommend that the author remove the word "also".**

Thank you for your patient and detailed comments; we have made revisions according to your suggestions, in Lines 29-31, we added:

"The drug carrier systems with photothermal effect can promote the killing of cancer cells and help overcome drug resistance through heat stress"

And Lines 74-76

"Therefore, utilizing the synergistic effect of chemotherapy and hyperthermia has become an important direction for the development of new drugs."

**（2）Some unclear expressions need clarification. In the introduction part, "Therefore, more precise, minimally invasive, and effective treatments are urgently needed, and the combination of new treatments with radiotherapy, chemotherapy, and other therapies is beginning to receive attention". What other treatments? The article does not mention, please clarify.**

**Please explain specifically what is meant by "Namely…** **given that cancer cells exhibit different susceptibility to cell death induced by heat compared with normal cells."**

We apologize for any lack of clarity in our initial explanation, we have accordingly made the following adjustments and additions:

In Lines 63-67

"Therefore, more precise, minimally invasive, and effective treatments are urgently needed, and the combination of new treatments (e.g., heat therapy and immunotherapy) with standard treatments (radiotherapy, chemotherapy, and surgical treatment) is beginning to receive attention."

In Lines 68-73

"In general, cancer cells exhibit different susceptibility to cell death induced by heat compared with normal cells. Exposure to the same thermal dose (defined by exposure time and given temperature) is more likely to induce a cell death outcome in cancer cells. Therefore, appropriate heat stress can promote the killing of tumor cells without causing damage to normal cells."

**（3）Please pay attention to formatting and punctuation.**

**There are extra spaces in some locations. The presence or absence of spaces before and after mathematical symbols needs to be consistent, such as** **"T/C (%) ≤40%".There should not be a hyphen between** **"24-h" or "48-h".**

Thank you for your comments. We have checked the mathematical symbols, hyphens, and spaces throughout the text and corrected the above two types of problems.

Line 400: Changed from "at >40%" to "at > 40%".

Line 504: Changed from "24-h or 48-h" to "24 h or 48 h".

Line 658: Changed from "T/C (%) ≤40%" to "T/C (%) ≤ 40%".

**（4）It is recommended to give the statistical method used for the results.**

Thank you for your suggestions. We have provided a more detailed description in the Methods section, Lines 311-314:

Differences between groups were determined using the t-test. Student' s t-test was chosen when the two groups of samples have equal variances. If samples have unequal variances, Welch' s t-test was used.

**（5） The ruler format in Figure 1 is inconsistent, please modify it.**

Thanks for your meticulous comments. In Fig. 1A-1C, we used SEM imaging, while TEM imaging is used in Fig. 1D. The format of the ruler varies with different software, but considering the credibility of the pictures, we used the original output pictures without modifying the original scale style.

**（6）Some pictures do not provide the length of the** **scale bars, such as Figure 6 and 7, please add relevant information.**

We apologize for the oversight in our paper writing. We have supplemented the legends of Figures 6, 7, and 9.

**7. The conclusion is interesting, but lacks more intuitive data. It is suggested to give quantitative conclusions on the key results.**

Thank you for your valuable suggestions. We have made the following modifications to the important results in the original text:

Lines 535-537

"……and the inhibition rate of nPDA-cis balls without combined photothermal treatment after 48 h was 2.3 times higher than that of the cisplatin-only group (p < 0.001) (Fig. 5C)"

Lines 628-633, We have changed it into

"On day 10, the average tumor volume of nPDA-cis balls with NIR group was significantly smaller than cisplatin with NIR group (p < 0.01), and was only 30.04% that of cisplatin with NIR group. Besides, the average tumor volume of nPDA-cis balls group was only 71.47% that of cisplatin group. Apparently, nPDA-cis balls showed the best tumor inhibition with and without NIR laser irradiation, and they significantly outperformed cisplatin."

Lines 658-663

As a result, nPDA-cis balls with photothermal treatment showed the best therapeutic effect, the relative tumor proliferation rate T/C was 27.59%, and was statistically significant better than cisplatin with photothermal treatment group (p < 0.001) (Fig. 8D). nPDA-cis balls without photothermal treatment also showed statistically significant better therapeutic effect than cisplatin group (p < 0.01) (Fig. 8D).

**Review Editor, Comments to the Author:**

This manuscript is of initial interest to the Board but will require significant revisions before the potential publication. Please submit a revised version which will be reviewed again.

Meanwhile, the authors should further improve the graphic abstract (GA).

The size of your GA in the manuscript is correct, but it is incorrect (3.61 cm * 8.78 cm) in the individual document of GA. Please fix this problem and the authors can move the elements like HNSCC to a little bit right position so that the empty space on the right edge could be eliminated.

We are sorry that the drawing is not precise enough, and we have modified the GA which meets size (3.6 cm ×8.4 cm) requirements. The modified version of the individual document of GA looks like this:

1.	Zhao, J.C. and M.H. Stenzel. Entry of nanoparticles into cells: the importance of nanoparticle properties. Polymer Chemistry, 2018. 9(3): p. 259-272.

2.	Shang, L., K. Nienhaus, and G.U. Nienhaus, Engineered nanoparticles interacting with cells: size matters. Journal of Nanobiotechnology, 2014. 12: p. 11.

3.	Rennick, J.J., A.P.R. Johnston, and R.G. Parton, Key principles and methods for studying the endocytosis of biological and nanoparticle therapeutics. Nature Nanotechnology, 2021. 16(3): p. 266-276.

4.	Cao D., Ding J. Recent advances in regenerative biomaterials. Regenerative Biomaterials, 2022. 9: p. rbac098.

5.	Noronha, V., et al., Once-a-Week Versus Once-Every-3-Weeks Cisplatin Chemoradiation for Locally Advanced Head and Neck Cancer: A Phase III Randomized Noninferiority Trial. Journal of Clinical Oncology : Official Journal of the American Society of Clinical Oncology, 2018. 36(11): p. 1064-1072.

6.	Jhawar, S.R., M. Bonomi, and P.M. Harari, Treating Advanced Head and Neck Cancer When Cisplatin Is Not an Option. Journal of Clinical Oncology : Official Journal of the American Society of Clinical Oncology, 2021. 39(1).

7.	Li, Y., et al., Decomposable black phosphorus nano-assembly for controlled delivery of cisplatin and inhibition of breast cancer metastasis. J Control Release, 2021. 335: p. 59-74.

8.	Kuang, Y., et al., Dual-Stimuli-Responsive Multifunctional Gd(2)Hf(2)O(7) Nanoparticles for MRI-Guided Combined Chemo-/Photothermal-/Radiotherapy of Resistant Tumors. ACS Appl Mater Interfaces, 2020. 12(32): p. 35928-35939.

9.	Liu, J., et al., Redox/pH-responsive hollow manganese dioxide nanoparticles for thyroid cancer treatment. Front Chem, 2023. 11: p. 1249472.

10.	Tran, A.V., et al., Targeted and controlled drug delivery by multifunctional mesoporous silica nanoparticles with internal fluorescent conjugates and external polydopamine and graphene oxide layers. Acta Biomater, 2018. 74: p. 397-413.

11.	Li, Q., et al., Targeted therapy for head and neck cancer: signaling pathways and clinical studies. Signal Transduction and Targeted Therapy, 2023. 8(1): p. 31.

12.	Yue, Y. and X. Zhao, Melanin-Like Nanomedicine in Photothermal Therapy Applications. Int J Mol Sci, 2021. 22(1).

13.	Yazdi, M.K., et al., Polydopamine Biomaterials for Skin Regeneration. ACS Biomater Sci Eng, 2022. 8(6): p. 2196-2219.

14.	He, X., et al., Polydopamine, harness of the antibacterial potentials-A review. Mater Today Bio, 2022. 15: p. 100329.

15.	Alfieri, M.L., et al., Polydopamine at biological interfaces. Adv Colloid Interface Sci, 2022. 305: p. 102689.

16.	Zheng, P., B. Ding, and G. Li, Polydopamine-Incorporated Nanoformulations for Biomedical Applications. Macromol Biosci, 2020. 20(12): p. e2000228.
